# MMP8 Promotes NETosis in Gestational Diabetes Mellitus

**DOI:** 10.3390/antiox15080955

**Published:** 2026-07-30

**Authors:** Nan Li, Tong Zhou, Kun Yang, Wen-Jun Yang, Gui Yang, Yong-Wei Duan, Ying Yang, Huan-Yu Liu, Song-Mei Liu

**Affiliations:** 1 Department of Clinical Laboratory, Center for Gene Diagnosis and Program of Clinical Laboratory, Zhongnan Hospital of Wuhan University, Wuhan 430071, China; 2 Department of Pharmacy, Zhongnan Hospital of Wuhan University, Wuhan 430071, China; 3 Department of Obstetrics, Zhongnan Hospital of Wuhan University, Wuhan 430071, China; 4 Clinical Research Center for Prenatal Diagnosis and Birth Health of Hubei Province, Wuhan 430071, China

**Keywords:** gestational diabetes mellitus, neutrophil extracellular traps, MMP8

## Abstract

**Background:** Neutrophil extracellular traps (NETs) have been known to be involved in the gestational diabetes mellitus (GDM), but the underlying role remains poorly understood. **Methods:** We conducted an integrated analysis of bulk RNA-seq data, single-cell transcriptomic sequencing (scRNA-seq) data, clinical laboratory findings, and cell models to identify hub genes linked to NET formation (NETosis) and to elucidate how NETs contribute to placental injury in GDM. **Results:** We found that circulating NET levels were increased in pregnant women with GDM both under fasting conditions and following an oral glucose tolerance test (OGTT). Primary neutrophils isolated from healthy pregnant women produced more NETs upon high-glucose stimulation in vitro. The mRNA expression of *PADI4*, a key regulator of NETosis, was upregulated and positively correlated with *MMP8* mRNA in patients with GDM. Inhibition of MMP8 suppressed intracellular reactive oxygen species (ROS) generation and attenuated NETosis in neutrophils. scRNA-seq analysis of placental tissues from patients with GDM identified a neutrophil subset with higher *PADI4* expression. In vitro stimulation with NETs induced functional impairment of HTR8/SVneo cells. **Conclusions:** We uncovered that MMP8, a novel NETosis-promoting molecule, is a promising target for GDM intervention.

## 1. Introduction

Gestational diabetes mellitus (GDM) is one of the most prevalent metabolic complications during pregnancy, affecting a substantial proportion of pregnant women worldwide. The incidence of GDM is rising owing to lifestyle changes and advanced maternal age [1]. GDM is characterized by systemic low-grade inflammation, including immune cell activation [2,3] and dysregulated inflammatory cytokine production [4,5]. This inflammatory state not only impairs maternal glucose metabolism but also contributes to placental dysfunction [6]. Furthermore, it may result in a range of long-term adverse outcomes in offspring, including increased risk of type 2 diabetes [7,8], obesity [8], and metabolic syndrome [9]. Despite advances in clinical screening and glycemic management, the molecular mechanisms underlying GDM-associated inflammation and metabolic dysfunction remain to be fully elucidated.

Neutrophil extracellular traps (NETs) are web-like structures composed of decondensed chromatin and granular proteins released by activated neutrophils [10]. NETs have emerged as critical mediators of inflammation in various metabolic disorders, including diabetes [11,12] and obesity [13]. While NETs play important roles in immune defense [14], excessive NETosis contributes to endothelial dysfunction [15], oxidative stress [16], and immune dysregulation [17], all of which are characteristic pathological features of GDM. Accumulating evidence indicates that NETs and NET-related markers are elevated in the peripheral blood [18,19] and placental tissues [20] of patients with GDM, suggesting a potential role for NETs in the pathogenesis of GDM.

As a vital gestational organ featuring intricate and dynamic gene regulatory networks, the placenta plays a central role in modulating maternal immunity throughout pregnancy [21,22]. Neutrophils are one of the major innate immune cell populations detected in the placenta [23], but their functional heterogeneity and the specific subsets contributing to GDM-associated inflammation have not been fully defined. Single-cell transcriptomic sequencing (scRNA-seq) has emerged as a powerful tool to dissect cellular heterogeneity and characterize cell-type-specific functional signatures in complex tissues [24,25]. The widespread application of scRNA-seq has provided novel insights into pregnancy-related disorders [26].

The present study aimed to screen hub genes correlated with GDM using a bulk RNA-seq dataset and to evaluate the expression of these hub genes and NET levels in clinical samples. In addition, further experiments were conducted to validate the roles of hub genes in the NET formation (NETosis), and an scRNA-seq dataset was analyzed to characterize the transcriptional signatures of neutrophil subsets in GDM placentas. Moreover, HTR8/SVneo cells were used to investigate the effects of NETs on extravillous trophoblasts (EVTs). Overall, we identified MMP8 as a novel regulator of NETosis and a potential therapeutic target for GDM.

## 2. Methods

### 2.1. Bulk RNA-Seq Dataset Analysis

The GSE92772 dataset was obtained from the Gene Expression Omnibus (GEO) database. Differential expression analysis was performed using the limma, DESeq2, and edgeR packages in R (v4.3.2). Differentially expressed genes (DEGs) were identified using the criteria of |log_2_FC| ≥ 1 and *p* < 0.05, and overlapping DEGs were visualized using a Venn diagram (https://bioinformatics.psb.ugent.be/webtools/Venn/, accessed on 30 December 2024). Gene Ontology (GO) enrichment analysis was performed using DAVID (https://davidbioinformatics.nih.gov/, accessed on 30 December 2024), and a protein-protein interaction (PPI) network was constructed using the STRING database (https://string-db.org/, accessed on 30 December 2024). Hub genes were identified from the PPI network using the cytoHubba, MCODE, and CytoNCA plugins in Cytoscape (v3.7.1). Gene set enrichment analysis (GSEA) was conducted using clusterProfiler (v4.9.0) with gene sets obtained from the Molecular Signatures Database (MSigDB). For GSEA, *p* values were adjusted using the Benjamini–Hochberg method, and an adjusted *p* < 0.05 was considered statistically significant.

### 2.2. scRNA-Seq Dataset Analysis

GSE267340 was obtained from GEO and processed using Seurat (v5.3.1) [27]. Cells with fewer than 200 or more than 6000 detected genes, or with more than 15% mitochondrial gene expression, were excluded. Cells presumed to be doublets were filtered out using scDblFinder (v1.24.8) [28], and ambient RNA contamination was eliminated using celda’s decontX (v1.26.0) [29]. Then, gene expression matrices were normalized using LogNormalize. Additionally, unsupervised clustering and Uniform Manifold Approximation and Projection (UMAP) visualization were conducted with a clustering resolution of 0.5 (Supplementary Figure S1). Cell types were annotated according to canonical marker genes (Supplementary Figure S2). Furthermore, differential expression analysis was performed using FindMarkers. DEGs were defined as genes with an adjusted *p* < 0.05 and |log2 fold change| > 0.25, and *p* values were adjusted using the Bonferroni method. Subsequently, trajectory inference was performed using Slingshot (v2.10.0) [30]. Finally, cell–cell communication was analyzed using CellChat (v2.2.0) [31]. Aggregated networks and key signaling pathways were analyzed via aggregateNet and computeCommunProbPathway, respectively.

### 2.3. Sample Collections

Pregnant women were recruited from the Zhongnan Hospital of Wuhan University (Wuhan, Hubei, China). GDM was diagnosed according to the International Association of Diabetes and Pregnancy Study Groups (IADPSG) criteria, based on a 75 g oral glucose tolerance test (OGTT) performed at 24–28 weeks of gestation, with any of the following plasma glucose values: fasting plasma glucose ≥ 5.10 mmol/L, 1 h glucose ≥ 10.00 mmol/L, or 2 h glucose ≥ 8.50 mmol/L [32]. Fresh peripheral blood samples were collected during OGTT (24–28 weeks of gestation), and placental tissues were collected at delivery (Supplementary Figure S3). The GDM group and the control group were matched for maternal age and gestational age. The exclusion criteria were pregestational diabetes, autoimmune disease, acute infection, and cancers. Demographic and clinical information of pregnant women and newborns was collected from medical records. This study was reviewed and approved by the Medical Ethics Committee of Zhongnan Hospital of Wuhan University in accordance with the Declaration of Helsinki (Approval No. 2025112K). Informed consent was obtained from all participants prior to sample collection.

### 2.4. Isolation of Neutrophils and NET Collection

Neutrophils were isolated from peripheral blood using a peripheral blood neutrophil separation kit (Solarbio, Beijing, China) according to the manufacturer’s instructions. The isolated neutrophils were cultured in RPMI-1640 (Procell, Wuhan, China) medium with 10% FBS (Vazyme, Nanjing, China).

Freshly isolated neutrophils from healthy donors were stimulated with 500 nM phorbol 12-myristate 13-acetate (PMA, MCE, Monmouth Junction, NJ, USA) for 4 h. The layer of NETs and neutrophils adhered to the bottom was collected and centrifuged at 450× *g* for 10 min. Then, the cell-free NET-rich supernatant was obtained and centrifuged at 12,000× *g* for 10 min at 4 °C. Finally, the supernatant was discarded, and all pelleted NETs were resuspended in cold PBS (Servicebio, Wuhan, China). The DNA concentration of NETs was subsequently quantified with the Qubit™ dsDNA Assay Kit (Invitrogen, Carlsbad, CA, USA).

### 2.5. Enzyme-Linked Immunosorbent Assay (ELISA)

Plasma NET levels were determined using human NET ELISA kit (Jianglai, Shanghai, China) according to the manufacturer’s instructions. The absorbance at 450 nm was obtained with an automatic microplate reader (PerkinElmer, Waltham, MA, USA).

### 2.6. Quantitative Real-Time PCR (qPCR)

Total RNA was extracted using TRIzol (Invitrogen, Carlsbad, CA, USA), and cDNA was synthesized from RNA using the HiScript^®^ III RT SuperMix (Vazyme, Nanjing, China). mRNA expression was quantified by qPCR (Vazyme, Nanjing, China), calculated via the 2^−ΔΔCT^ method, and normalized to β-actin. Primer sequences are listed in Supplementary Table S1.

### 2.7. Western Blot

Cell proteins were extracted with RIPA buffer (Biosharp, Beijing, China) and quantified using a BCA assay kit (Solarbio, Beijing, China). Proteins were separated by SDS-PAGE (Servicebio, Wuhan, China) and transferred to PVDF membranes (Millipore, Burlington, MA, USA). After blocking, membranes were incubated with Bax (1:1000), Bcl2 (1:1000) and β-actin (1:3000) primary antibodies overnight at 4 °C, followed by HRP-conjugated secondary antibodies (1:10,000). Protein bands were visualized with a highly sensitive imaging system (e-BLOT, Shanghai, China) and analyzed by Image J. All antibodies used in this study were purchased from Proteintech (Wuhan, China), unless otherwise specified.

### 2.8. Co-Immunoprecipitation (Co-IP)

Co-IP was conducted using the Immunoprecipitation Kit with Protein A+G Magnetic Beads (Beyotime, Shanghai, China) following the manufacturer’s instructions. The cell lysates were incubated with the anti-MMP8 antibody (1:400) or anti-PADI4 antibody (1:250) conjugated on magnetic beads overnight at 4 °C. Next, the beads were washed five times with lysis buffer, eluted, and mixed with loading buffer for Western blot analysis.

### 2.9. Immunofluorescence (IF) Staining

Primary neutrophils were seeded on poly-l-lysine-coated coverslips (Biosharp, Beijing, China). Cells were fixed, permeabilized and blocked, then incubated with anti-citH3 (1:200, Abcam, Cambridge, UK) and anti-MPO (1:200) primary antibodies overnight at 4 °C. After PBS washing, cells were incubated with fluorescent secondary antibodies, and nuclei were stained with DAPI (Biosharp, Beijing, China). Fluorescence images were acquired by an inverted fluorescence microscope (Olympus, Tokyo, Japan).

### 2.10. Immunohistochemistry (IHC) Staining

Placental tissues were fixed, dehydrated and embedded in paraffin. Tissue sections were incubated with citH3 (1:200) and MPO (1:200) primary antibodies overnight at 4 °C. After PBS washing, samples were incubated with the corresponding secondary antibodies. Then, sections were stained with DAB (Proteintech, Wuhan, China) and hematoxylin (Proteintech, Wuhan, China), and histological images were observed under an Olympus light microscope.

### 2.11. Determination of ROS Assay

Intracellular reactive oxygen species (ROS) levels were measured using a ROS assay kit (Yeasen, Shanghai, China). Cells were incubated in RPMI-1640 medium containing DCFH-DA and Hoechst 33342 (Yeasen, Shanghai, China) in the dark. After PBS washing, fluorescence images were captured using an Olympus inverted fluorescence microscope.

### 2.12. Cell Culture

Primary neutrophils were maintained in RPMI-1640 medium (Procell, Wuhan, China) supplemented with 10% FBS (Vazyme, Nanjing, China) and 1% penicillin–streptomycin antibiotics (Biosharp, Beijing, China). Neutrophils isolated from healthy pregnant women were stimulated with 33.3 mmol/L high glucose (HG) and 5.5 mmol/L low glucose (LG) for 24 h to explore the effects of HG on NETosis. Neutrophils isolated from healthy pregnant women and pregnant women with GDM were cultured without treatment for 24 h to evaluate spontaneous NETosis. Neutrophils isolated from healthy donors were stimulated with 500 nM PMA for 4 h with or without MMP8 inhibitor-1 (M8I, MCE, Monmouth Junction, NJ, USA) to clarify the regulatory role of M8I in NETosis; Neutrophils from healthy donors were incubated with 0–100 ng/mL NETs for 2–4 h to investigate the effects of NETs on *MMP8* and *LTF* (Supplementary Figure S4).

Human chorionic trophoblast cells (HTR8/SVneo, Cell Bank, Chinese Academy of Sciences, Shanghai, China) were maintained in RPMI-1640 medium supplemented with 10% FBS and 1% antibiotic penicillin–streptomycin at 37 °C. To explore the effects of HG and NETs on trophoblasts, HTR8/SVneo cells were maintained in LG or HG medium (with or without 100 ng/mL NETs) for 24 h.

### 2.13. CCK-8 Proliferation Assay

Cell viability was assessed using the Cell Counting Kit-8 (CCK-8) reagent (Biosharp, Hefei, China) following the manufacturer’s protocol. HTR8/SVneo cells were seeded in 96-well plates and cultured overnight, followed by the addition of different interventions. At 0 h, 24 h, 48 h, and 72 h, absorbance was detected at 450 nm with an automatic microplate reader.

### 2.14. Wound Healing Assay

The wound healing assay was used to assess the migratory capacity of HTR8/SVneo. The cells were cultured in low-serum medium, and wound area images were captured at 0 h, 24 h, and 48 h after scratching. Quantitative analysis of cell migration was performed using Image J (v 1.54p).

### 2.15. Transwell Assay

Transwell assays were performed using 8 μm pore inserts (Corning, Union City, CA, USA). HTR8/SVneo cells were seeded in the upper chamber, while the lower chamber was supplemented with or without NETs. After 48 h incubation, migrated cells were fixed, stained with crystal violet, and quantified using an Olympus microscope.

### 2.16. Cell Apoptosis Assay

Cell apoptosis was assessed using an Annexin V-FITC/PI apoptosis kit (Vazyme, Nanjing, China) according to the manufacturer’s instructions. After trypsinization and PBS washing, cells were resuspended in binding buffer, stained with Annexin V-FITC and PI in the dark, and apoptosis was detected by a Beckman Coulter CytoFLEX flow cytometer.

### 2.17. Statistical Analysis

Statistical analysis was performed using SPSS 27.0, Origin 2025, GraphPad Prism 10.0, and R (v4.3.2). Student’s *t*-test and one-way ANOVA were applied to compare differences among normally distributed data. The Mann-Whitney U test was used for skewed data. The paired *t*-test was adopted for pairwise comparisons. The Chi-square test was utilized to analyze categorical variables. Pearson’s and Spearman’s correlation coefficients were used to evaluate associations between variables. Statistical significance was defined as *p* < 0.05.

## 3. Results

### 3.1. Identification of Hub Genes in the Peripheral Blood of GDM Patients

**Figure 1:**
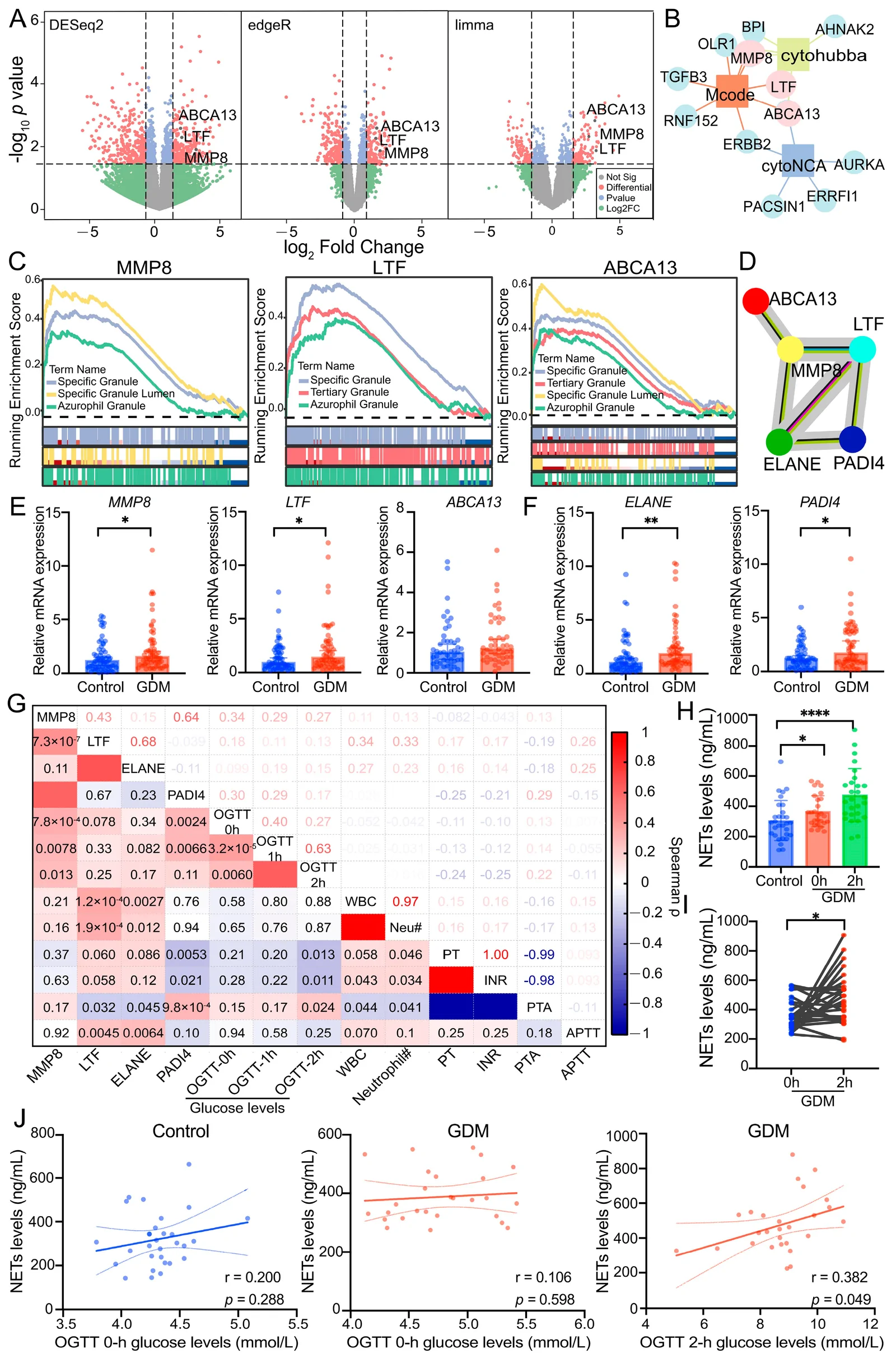
Identification and validation of candidate hub genes in peripheral blood leukocytes from women with GDM. (**A**) Volcano plot showing differentially expressed genes (DEGs) in GDM patients. (**B**) Identification of candidate hub genes via MCODE, CytoHubba, and CytoNCA algorithms. (**C**) Gene set enrichment analysis (GSEA) illustrating significant enrichment of candidate hub genes in granule-associated gene sets. (**D**) PPI network visualizing protein-protein interactions between candidate hub genes and critical NETosis enzymes (ELANE and PADI4). (**E**) qPCR results showing upregulated mRNA expression of *MMP8* and *LTF* in peripheral blood leukocytes from GDM patients. (**F**) Bar chart presenting upregulation of *ELANE* and *PADI4* in peripheral blood leukocytes in GDM. (**G**) Correlation heatmap of hub genes and laboratory parameters. (**H**) Plasma NET levels were significantly elevated in GDM patients. (**I**) Paired scatter plot showing OGTT 2 h plasma NET levels were elevated compared with those at fasting in GDM. (**J**) Scatter plot showing the positive correlation between OGTT 2 h plasma NET levels and glucose levels in GDM. * *p* < 0.05, ** *p* < 0.01, **** *p* < 0.0001. Neu#: Absolute neutrophil count (10^9^/L).

DESeq2, edgeR, and limma identified 565, 330, and 218 DEGs, respectively (Figure 1A). A total of 90 shared DEGs were visualized using a Venn diagram (Supplementary Figure S5A). GO enrichment analysis revealed that the shared DEGs were significantly enriched in antibacterial humoral response, protein phosphorylation, mitotic cell cycle, and antimicrobial humoral immune response mediated by antimicrobial peptides. Cellular component analysis demonstrated significant enrichment in specific granule lumen and extracellular region (Supplementary Figure S5B), suggesting alterations in extracellular and granule-associated functions. Collectively, these findings indicate that immune activation and dysregulated extracellular granule-related processes may contribute to the pathogenesis of GDM. Three candidate hub genes (*ABCA13*, *MMP8*, and *LTF*) were identified from the PPI network (Supplementary Figure S5C) using the MCODE, CytoHubba, and CytoNCA algorithms (Figure 1B). GSEA results showed that their expression levels were positively correlated with granule-related functions (Figure 1C). Since neutrophil degranulation is necessary for NETosis [33], the upregulation of these candidate hub genes may be closely linked to NETosis in GDM. The PPI network further demonstrated that the three hub molecules interacted with core NETosis regulators, including PADI4 and ELANE (Figure 1D).

### 3.2. Validation of Bioinformatic Predictions in Clinical Samples

#### 3.2.1. Clinical Characteristics of the Study Participants

Table 1 summarizes the baseline characteristics of the study participants. Maternal age, gravidity, parity, mode of delivery, pregnancy complications, pregnancy comorbidities, and antenatal medication exposure were similar across groups (all *p* > 0.05). Gestational week at delivery was slightly lower in the GDM group (*p* = 0.039), which may be explained by clinical management involving earlier delivery in women with GDM. Women with GDM displayed higher prepregnancy weight and prepregnancy BMI but lower gestational weight gain (*p* = 0.002). OGTT glucose levels at different times were significantly increased in the GDM group (all *p* < 0.001), while peripartum fasting glucose levels did not differ between groups (*p* = 0.061). Glycemic management strategies and glycemic control status were only collected from the GDM group because routine blood glucose monitoring and intervention are not clinically required for pregnant women with normal glucose tolerance.

Regarding neonatal outcomes, birth anthropometric parameters, sex distribution, and transcutaneous bilirubin levels were comparable between neonates born to mothers with GDM and healthy controls (all *p* > 0.05). However, neonatal blood glucose was reduced in infants from GDM pregnancies (*p* = 0.048). The incidences of macrosomia and fetal distress did not differ between the two groups.

Multiple laboratory parameters differed significantly between the two groups, as presented in Supplementary Table S2. For hematological parameters, hemoglobin (HGB), mean corpuscular hemoglobin (MCH), and mean corpuscular hemoglobin concentration (MCHC) were significantly elevated in women with GDM (*p* = 0.042, *p* = 0.037, *p* = 0.045, respectively). Regarding coagulation parameters, D-dimer and prothrombin time activity (PTA) were significantly higher in the GDM group (*p* = 0.019 and *p* = 0.016), whereas prothrombin time (PT) and international normalized ratio (INR) were slightly lower compared with controls (*p* = 0.014, *p* = 0.010). Liver function tests revealed increased alanine aminotransferase (ALT) and gamma-glutamyl transferase (GGT) levels in women with GDM (*p* = 0.033, *p* = 0.048).

#### 3.2.2. Upregulation of *MMP8* and *LTF* in Peripheral Blood Leukocytes of Women with GDM

The mRNA expression of *LTF* (*p* = 0.042) and *MMP8* (*p* = 0.025) in peripheral blood leukocytes was increased in women with GDM compared with healthy controls (Figure 1E). Combined with the GSEA results, these findings indicated a potential association between *MMP8*, *LTF*, and neutrophil granule-related biological processes. We further detected the expression of *ELANE* and *PADI4*, two critical genes involved in NETosis. Figure 1F showed that the mRNA levels of *ELANE* (*p* = 0.002) and *PADI4* (*p* = 0.022) were higher in peripheral blood leukocytes from women with GDM than in controls. To test whether the expression of *MMP8* and *LTF* was associated with clinical laboratory parameters, we performed Spearman’s correlation analysis. *MMP8* expression was positively correlated with blood glucose levels (all *p* < 0.05). Meanwhile, *LTF* expression showed significant correlations with leukocyte counts and APTT (Figure 1G). Correlation analysis further revealed significant positive associations between *MMP8* and *LTF* (r = 0.43, *p* = 7.3 × 10^−7^), *MMP8* and *PADI4* (r = 0.64, *p* < 1.0 × 10^−7^), as well as *LTF* and *ELANE* (r = 0.68, *p* < 1.0 × 10^−7^) (Figure 1G). Taken together, these results support a potential link between *MMP8*/*LTF* expression and NETosis in GDM.

#### 3.2.3. High Glucose Promotes NETosis in GDM

**Figure 2:**
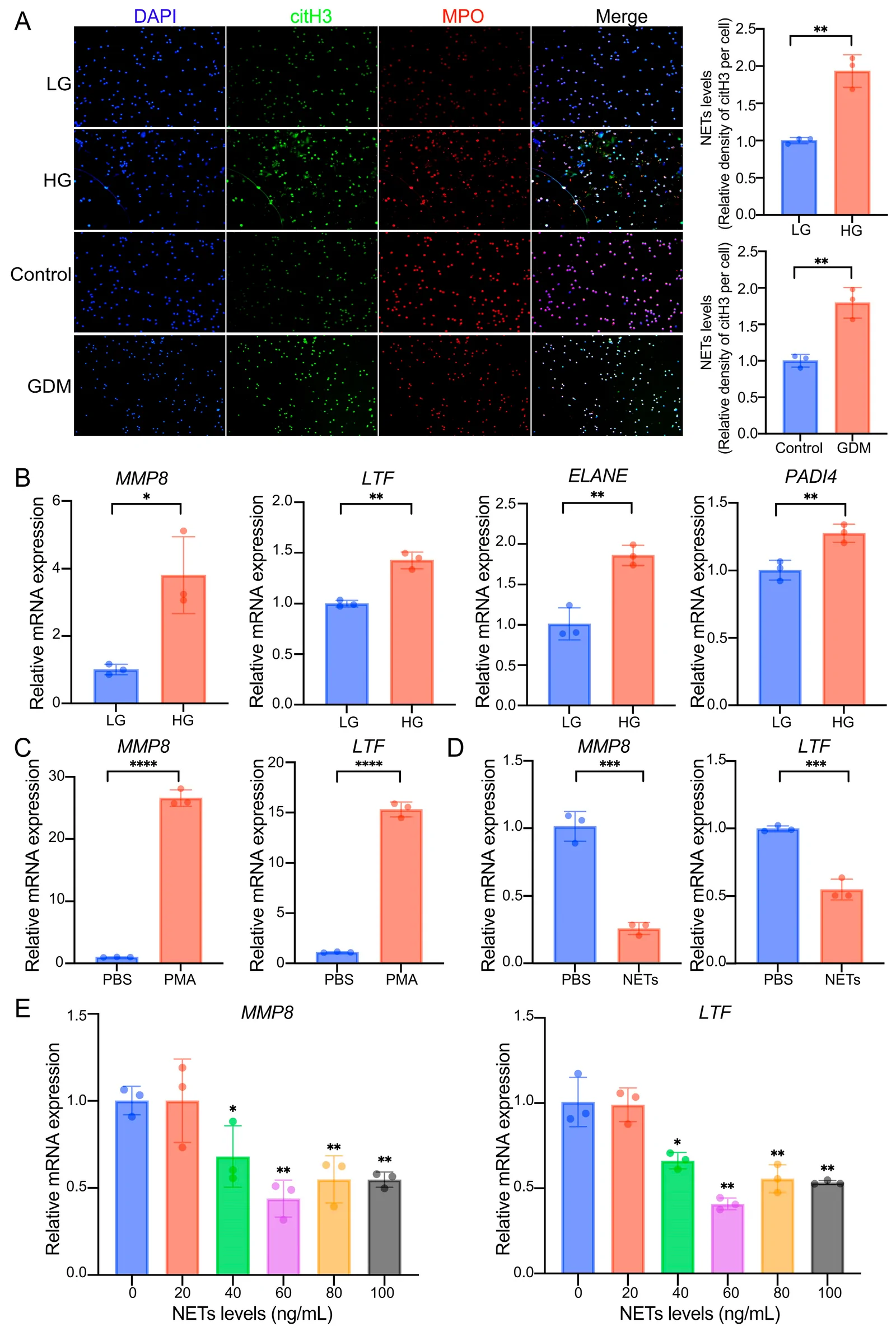
PMA and NETs Regulate *MMP8* and *LTF* Expression in Primary Neutrophils. (**A**) Immunofluorescence staining showing myeloperoxidase (MPO, red, neutrophil marker) and citrullinated histone H3 (citH3, green, NET-specific marker) in neutrophils (DAPI, blue, nuclear marker). Bar graph demonstrating the quantitative analysis of citH3 density in Control vs. GDM groups and LG vs. HG groups. (**B**) qPCR results showing HG upregulated the expression of *MMP8*, *LTF*, *ELANE* and *PADI4* in primary neutrophils. (**C**) Bar graph presenting the upregulation of *MMP8* and *LTF* expression by PMA in primary neutrophils. (**D**) The bar graph presenting NETs (100 ng/mL, 24 h) downregulated the expression of *MMP8* and *LTF* in primary neutrophils. (**E**) Bar graph showing NETs (40–100 ng/mL, 4 h) downregulated the expression of *MMP8* and *LTF* in primary neutrophils. LG, low glucose (5.5 mmol/L); HG, high glucose (33.3 mmol/L). * *p* < 0.05, ** *p* < 0.01, *** *p* < 0.001, **** *p* < 0.0001.

To investigate the potential association between NETosis and GDM, plasma samples collected from women with GDM and healthy controls during the OGTT (24~28 weeks) were used for NET detection. Circulating NET levels were higher in the GDM group than in the control group (340.9 [290.3, 449.9] vs. 289.3 [208.7, 374.1], *p* = 0.023) (Figure 1H). In addition, within the GDM group, circulating NET levels after oral glucose challenge were significantly higher than fasting levels (446.3 [347.1, 556.1] vs. 340.9 [290.3, 449.9], *p* = 0.029) (Figure 1I). Correlation analysis was further performed to evaluate the relationship between blood glucose and NET levels (Figure 1J). No significant correlation was found between fasting plasma glucose and NET levels in either the control group or the GDM group (*p* = 0.288, *p* = 0.598). Notably, OGTT 2 h plasma glucose levels exhibited a positive correlation with NET levels in the GDM group (r = 0.382, *p* = 0.049). Next, we isolated neutrophils from peripheral blood for in vitro culture and further explored the association between glucose and NETosis using immunofluorescence staining. Neutrophils isolated from women with GDM exhibited markedly enhanced spontaneous NETosis relative to those from healthy controls (Figure 2A). In addition, high glucose significantly promoted NETosis in primary neutrophils (Figure 2A). Consistently, high glucose also upregulated the mRNA expression of *MMP8*, *LTF*, *PADI4*, and *ELANE* in primary neutrophils (Figure 2B).

#### 3.2.4. PMA and NETs Regulate *MMP8* and *LTF* Expression in Primary Neutrophils

To investigate the relationship between *MMP8*, *LTF*, and NETosis, primary neutrophils from healthy donors were treated with PMA, a classical inducer of NETosis. After stimulation with 500 nM PMA for 4 h, the mRNA expression of *MMP8* and *LTF* in neutrophils were significantly upregulated (Figure 2C), which was consistent with the findings in peripheral blood. To further verify whether the upregulation of *MMP8* and *LTF* was triggered by NETs, primary neutrophils were incubated with exogenous NETs (100 ng/mL) for 24 h. In contrast to PMA stimulation, NET treatment resulted in marked downregulation of *MMP8* and *LTF* expression (Figure 2D). Given these opposing results, we propose that elevated NET levels might in turn inhibit *MMP8* and *LTF* expression. Primary neutrophils were then exposed to different concentrations of exogenous NETs (0–100 ng/mL) for 2 h and 4 h, respectively. Dose-dependent morphological alterations were observed in neutrophils following treatment with NETs (Supplementary Figure S6). At NET concentrations ≥ 40 ng/mL, neutrophils exhibited cell shrinkage, deformation, blurred cell boundaries, and membrane disruption. Significant downregulation of *MMP8* and *LTF* expression was also detected at 40 ng/mL NETs (Figure 2E). Collectively, these findings indicate that increased expression of *MMP8* and *LTF* in GDM may contribute to NETosis, while elevated NET levels may exert a negative feedback effect on *MMP8* and *LTF* expression.

#### 3.2.5. Inhibition of MMP8 Attenuates NETosis

PADI4 is a core enzyme that catalyzes histone citrullination and drives NETosis [34]. Given that *PADI4* mRNA expression showed a stronger positive correlation with *MMP8* mRNA expression in peripheral blood, we selected MMP8 as the research target. Since the technical challenges associated with genetic manipulation of primary neutrophils [35,36,37], we employed the MMP8 inhibitor-1 (M8I) for functional validation in vitro. However, inhibitor may inevitably involve potential off-target effects. To assess this possibility, we examined the expression of other MMP family members. No inhibitory effects of M8I on *MMP1* or *MMP9* were observed, whereas *MMP2* and *MMP13* were barely detectable in primary human neutrophils (Supplementary Figure S7).

**Figure 3:**
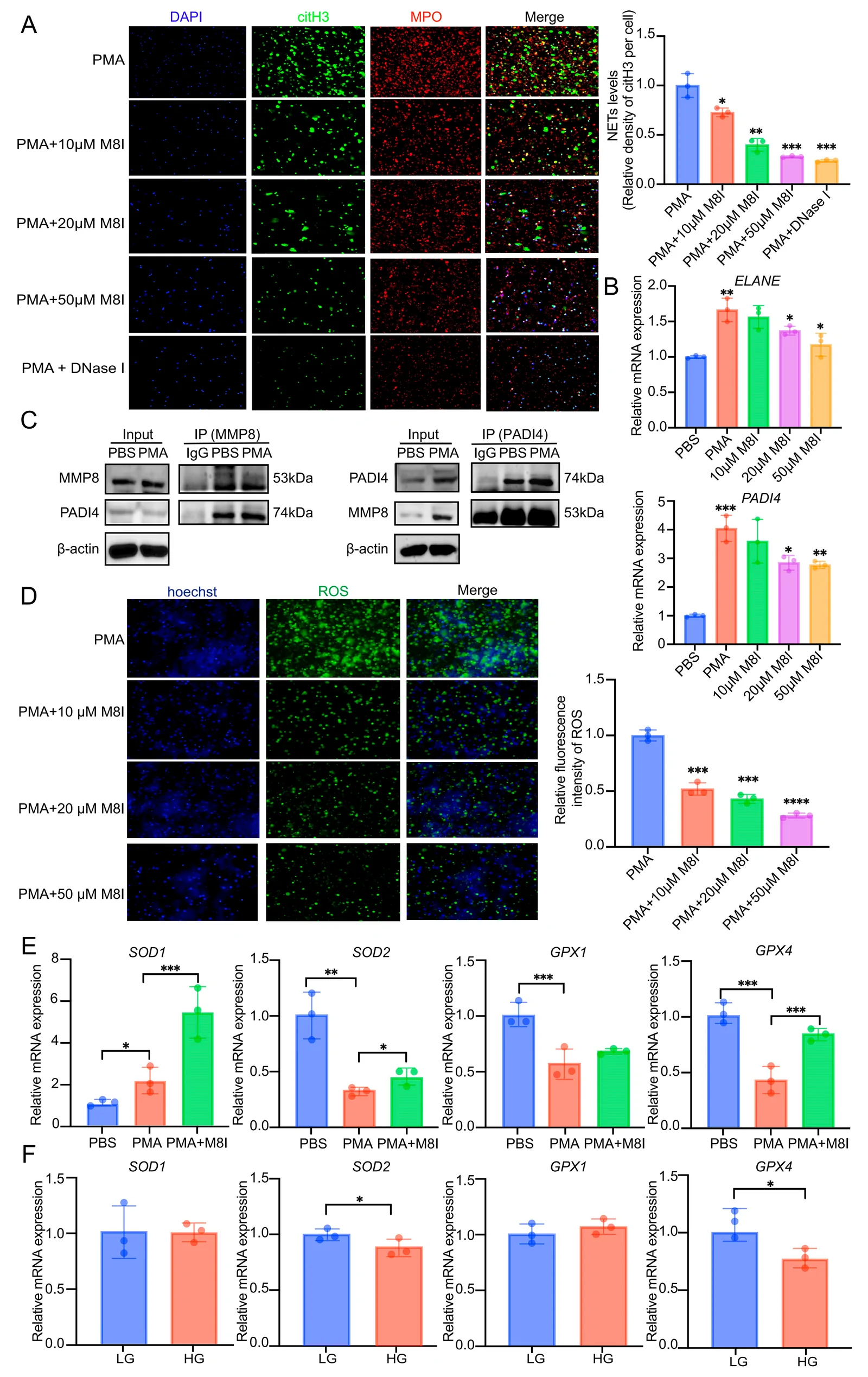
MMP8 Inhibitor Attenuates NETosis and Reduces ROS Production. (**A**) Representative immunofluorescence staining images of primary neutrophils treated with PMA alone, PMA combined with MMP8 inhibitor-1 (M8I), or PMA plus DNase I (positive control for inhibition of NETosis). Neutrophils were co-stained with DAPI (blue), citrullinated histone H3 (citH3, green) and myeloperoxidase (MPO, red). (**B**) qPCR results showing M8I downregulated the expression of *ELANE* and *PADI4* in PMA treated neutrophils. (**C**) Co-immunoprecipitation (Co-IP) showing a potential interaction between MMP8 and PADI4 in primary neutrophils treated with PBS or PMA for 4 h. (**D**) Immunofluorescence staining (Hoechst: blue, ROS: green) and quantitative analysis showing that treatment with M8I dose-dependently reduced intracellular ROS levels in PMA stimulated neutrophils. (**E**) Bar graph showing PMA stimulation significantly downregulated *SOD2* and *GPX4* expression, which was reversed by M8I treatment. (**F**) qPCR results showing HG downregulated *SOD2* and *GPX4* expression in primary neutrophils. LG, low glucose (5.5 mmol/L); HG, high glucose (33.3 mmol/L). * *p* < 0.05, ** *p* < 0.01, *** *p* < 0.001, **** *p* < 0.0001.

Primary neutrophils were cultured in vitro with M8I for 2 h, followed by PMA stimulation to trigger NETosis. Immunofluorescence results showed that M8I markedly reduced PMA-induced NETosis in a dose-dependent manner (Figure 3A). M8I also partially downregulated the mRNA levels of *ELANE* and *PADI4* (Figure 3B). Co-IP assays demonstrated that MMP8 physically interacted with PADI4 in neutrophils (Figure 3C). Given that NETosis is partly dependent on ROS production [38], we next evaluated the effects of M8I on intracellular ROS production in primary neutrophils following PMA stimulation. As shown in Figure 3D, PMA stimulation markedly increased intracellular ROS fluorescence intensity. Treatment with M8I significantly reduced PMA-induced intracellular ROS production in a dose-dependent manner. We next detected the mRNA expression of crucial antioxidant enzymes (*SOD1*, *SOD2*, *GPX1*, and *GPX4*) in neutrophils. M8I (20 μM) reversed the PMA-induced downregulation of *SOD2* and *GPX4*, whereas *SOD1* and *GPX1* were not significantly affected (Figure 3E). Consistent results were observed in the HG stimulation model. Compared with LG stimulation, HG stimulation downregulated *SOD2* and *GPX4* expression in neutrophils, with no significant changes in *SOD1* and *GPX1* (Figure 3F).

### 3.3. Validation of Activated Neutrophils in GDM Placentas by scRNA-Seq

**Figure 4:**
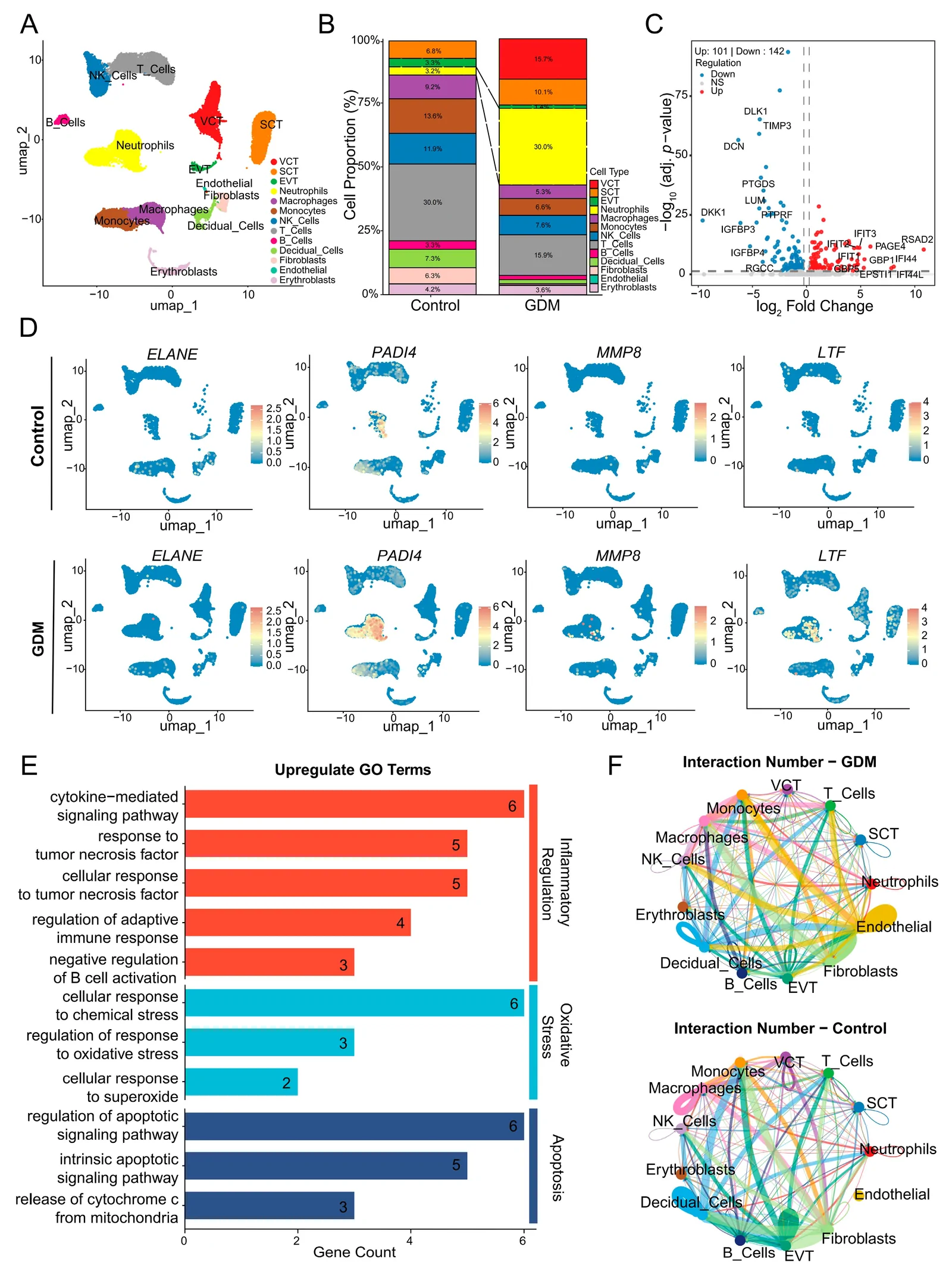
Dysregulation of Neutrophils in GDM Placentas. (**A**) Plot of cell type distribution in placentas. (**B**) Stacked bar charts showing the proportional distribution of all cell types in placental tissues from control and GDM. (**C**) Volcano plot of DEGs in GDM placental neutrophils. (**D**) Uniform Manifold Approximation and Projection (UMAP) visualizing the high expression of *ELANE*, *PADI4*, *MMP8*, and *LTF* in GDM placentas. (**E**) GO enrichment pathways of upregulated genes in GDM placental neutrophils. (**F**) CellChat analysis showing dysregulated intercellular interactions of GDM placental cell populations. VCT, villous cytotrophoblasts; SCT, syncytiotrophoblasts; EVT, extravillous trophoblasts.

Inspired by the clinical findings of elevated circulating NETs in women with GDM, we further explored whether activated neutrophils accumulate in GDM placentas and reshape placental immunity. A scRNA-seq dataset of GDM placentas was analyzed to characterize cell composition, gene expression profiles, and neutrophil populations. UMAP revealed that major placental cell types were clearly segregated into distinct clusters, including villous cytotrophoblasts (VCTs), syncytiotrophoblasts (SCTs), and extravillous trophoblasts (EVTs); immune cells (neutrophils, T cells, NK cells, B cells, monocytes, and macrophages); and stromal and vascular components (fibroblasts, endothelial cells, and decidual cells) (Figure 4A). Notably, the proportion of neutrophils was markedly higher in the GDM group, accounting for 30.0% of all cells, compared with 3.2% in healthy controls. In contrast, other immune cell subsets, such as T cells, macrophages, monocytes, and NK cells, were reduced in the GDM group (Figure 4B). Moreover, the proportion of EVTs were also reduced in GDM placentas (Figure 4B). As illustrated in the volcano plot (Figure 4C), a total of 243 DEGs were identified in neutrophils between GDM and control placentas. Consistent with our peripheral blood findings in women with GDM, feature plots revealed upregulation of *MMP8*, *LTF*, *ELANE*, and *PADI4* in GDM placental neutrophils (Figure 4D). In particular, *PADI4* exhibited the most significant upregulation, with expanded distribution and higher expression intensity across the neutrophil population in the GDM group. In GDM placental neutrophil populations, genes involved in immune regulation, oxidative stress, and apoptosis were upregulated (Figure 4E), while downregulated genes were involved in extracellular matrix organization, collagen metabolism, cell-substrate junction, and other related pathways (Supplementary Figure S8). To further explore the role of neutrophils in GDM placentas and their crosstalk with other cells, we performed intercellular communication analysis. As shown in Figure 4F, GDM placental neutrophils displayed extensive crosstalk with immune cells and EVTs. The neutrophil-centered intercellular communication network was markedly remodeled in GDM placentas.

#### 3.3.1. Features of Neutrophil Subsets in GDM Placentas

**Figure 5:**
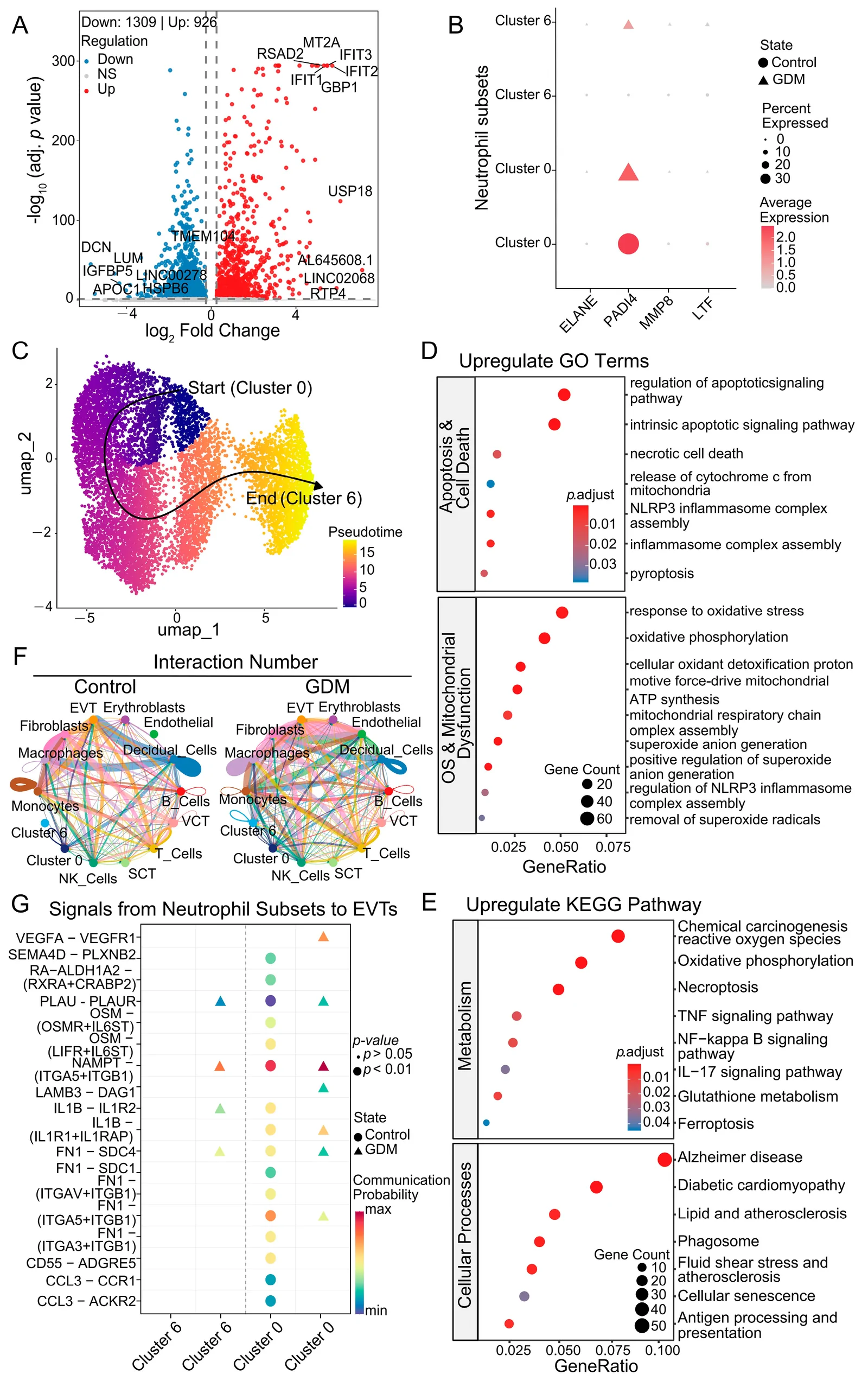
Features of Neutrophils Subsets in GDM Placentas. (**A**) Volcano plot showing DEGs between Cluster 6 and Cluster 0. (**B**) The expression of *MMP8*, *LTF*, *ELANE*, and *PADI4* in Cluster 6 and Cluster 0. (**C**) Pseudotime trajectory indicating Cluster 6 originated from Cluster 0. (**D**) GO enrichment pathways of upregulated genes in Cluster 6. (**E**) KEGG enrichment pathways of upregulated genes in Cluster 6. (**F**) Cell–cell communication network of placental cell populations by interaction number in control and GDM groups. (**G**) Ligand–receptor interaction between Cluster 6, Cluster 0 and EVTs in control and GDM groups. EVTs, extravillous trophoblasts.

Within the placental neutrophil population, we identified two distinct subsets (Cluster 0 and Cluster 6) with markedly different transcriptomic profiles (Figure 5A). Cluster 6 exhibited higher *PADI4* expression and was specifically expanded in GDM placentas. In contrast, Cluster 0 showed no significant differences in the expression of *ELANE*, *PADI4*, *MMP8*, and *LTF* (Figure 5B). Pseudotime trajectory analysis suggested a transition trajectory from Cluster 0 to Cluster 6 (Figure 5C). Genes upregulated in Cluster 6 were enriched in inflammatory responses, cell death, and oxidative stress pathways (Figure 5D). Signaling pathways including TNF, NF-κB, and IL-17 were significantly activated in Cluster 6 (Figure 5E). As shown in Supplementary Figure S9, genes downregulated in Cluster 6 were primarily associated with metabolic regulation, cell differentiation, and placental development. Moreover, PI3K-AKT, cAMP, TGF-β, and Wnt signaling pathways were notably suppressed in Cluster 6, which are closely associated with metabolic disorders and placental development in GDM. Furthermore, the interaction strength (Supplementary Figure S10A) and interaction number (Figure 5F) between neutrophil subsets and other cell types were remodeled in GDM placentas. CellChat analysis suggested that NAMPT/FN1 mediated signaling pathways contributed to the communication between Cluster 6 and EVTs (Figure 5G). In contrast, interactions between Cluster 0 and EVTs were diminished in GDM placentas (Figure 5G and Supplementary Figure S10B).

#### 3.3.2. Enhanced Neutrophil Accumulation and NETosis Promote Functional Dysfunction in HTR8/SVneo Cells

**Figure 6:**
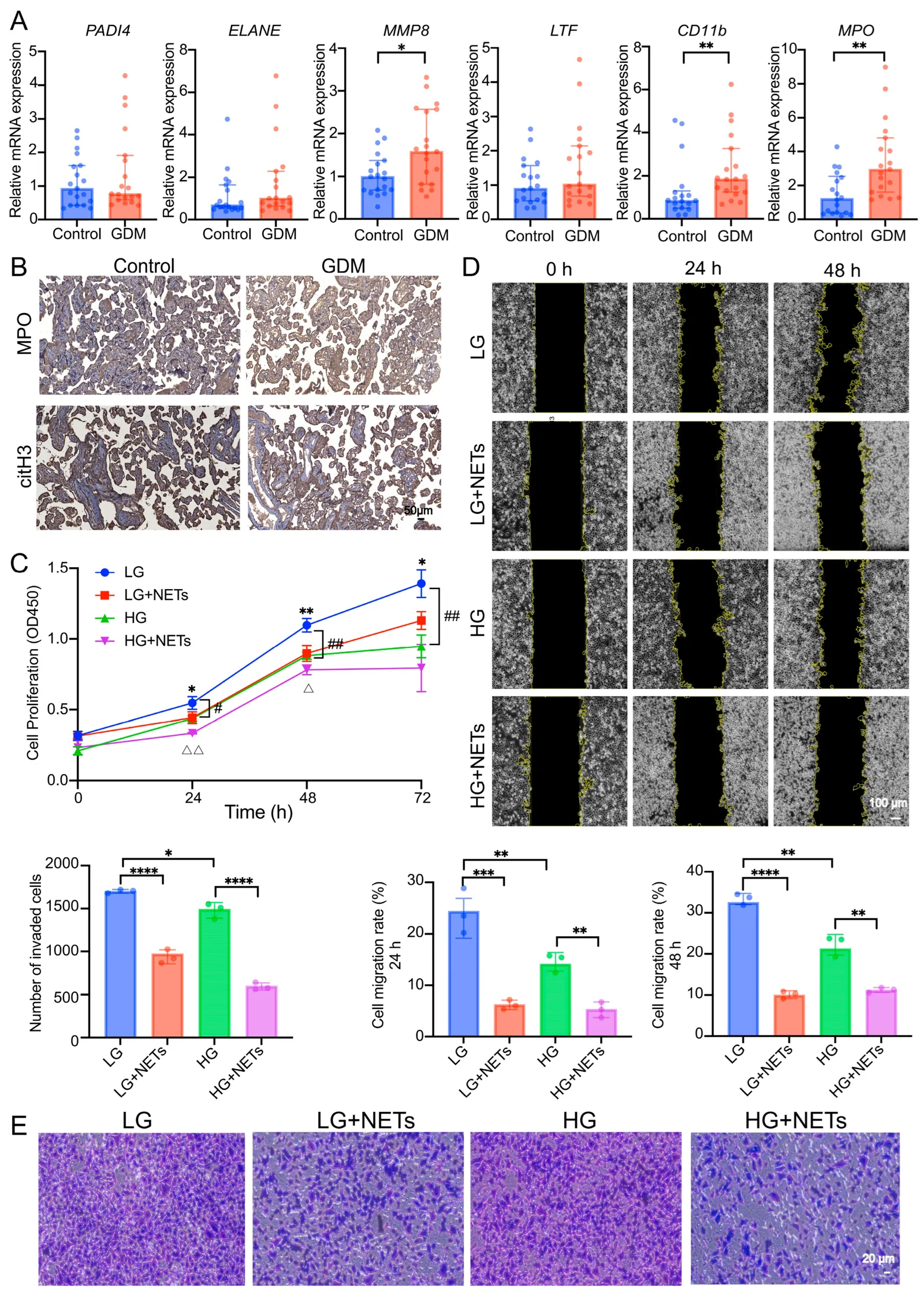
NETs promote HTR8/SVneo dysfunction. (**A**) qPCR results showing upregulation of *MMP8* and neutrophil markers (*CD11b* and *MPO*) in GDM placentas. (**B**) Immunohistochemical staining displaying elevation in NET markers (*MPO* and *citH3*) in GDM placental tissues. (**C**) CCK-8 results presenting HG and NETs inhibited HTR8/SVneo cell proliferation. *, LG vs. LG+NETs, * *p* < 0.05, ** *p* < 0.01; ^#^, LG vs. HG, ^#^ *p* < 0.05, ^##^ *p* < 0.01; ^Δ^, HG vs. HG+NETs, ^Δ^ *p* < 0.05, ^ΔΔ^ *p* < 0.01. (**D**) Wound healing assay indicating that NETs and HG inhibited the migration of HTR8/SVneo cells. (**E**) Transwell assay illustrating that NETs and HG inhibited the invasion of HTR8/SVneo cells. LG, low glucose (5.5 mmol/L); HG, high glucose (33.3 mmol/L). * *p* < 0.05, ** *p* < 0.01, *** *p* < 0.001, **** *p* < 0.0001.

**Figure 7:**
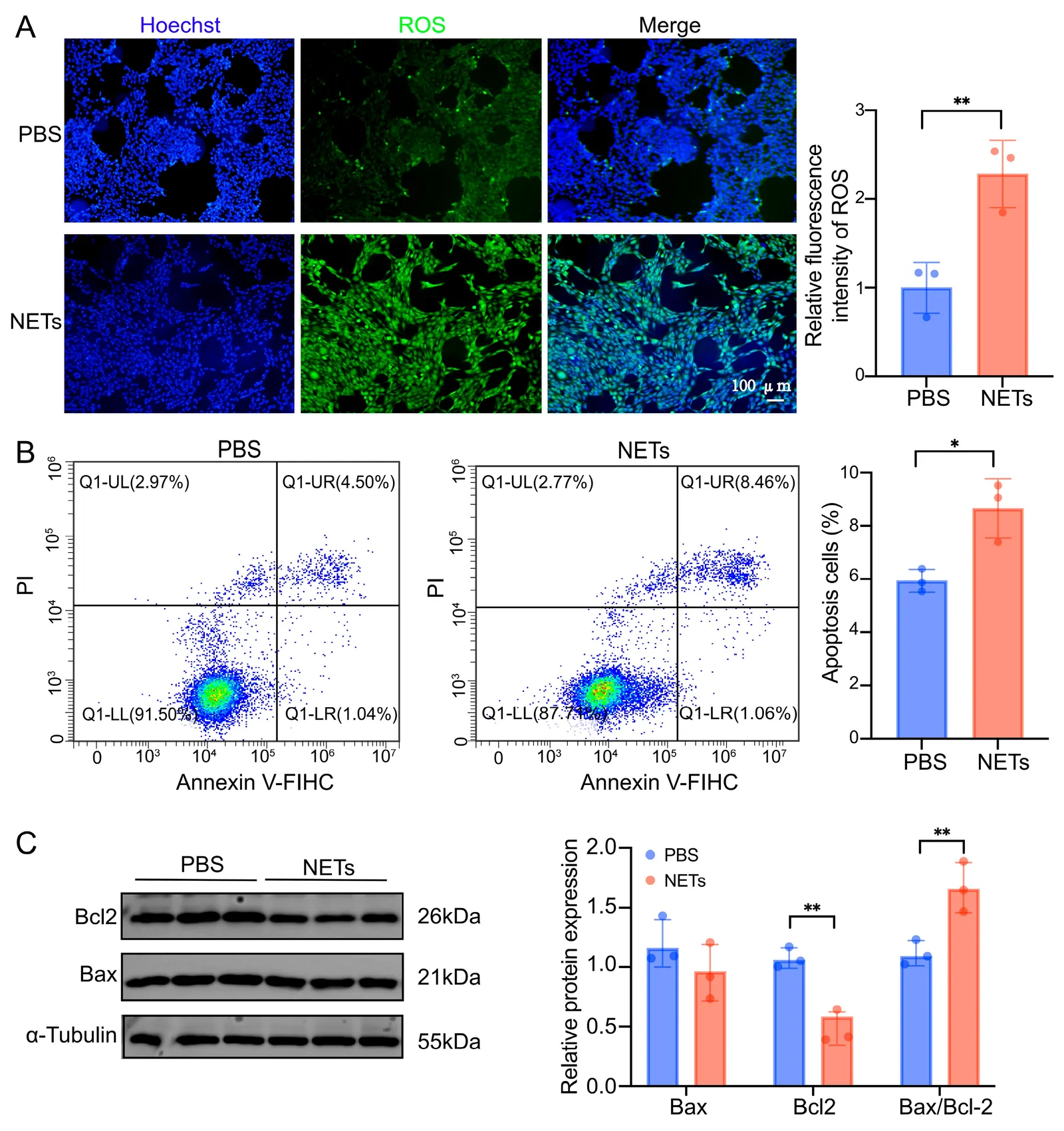
NETs induce ROS production and apoptosis of HTR8/SVneo cells. (**A**) Immunofluorescence staining images showing that NETs induced reactive oxygen species (ROS) production in HTR8/SVneo cells. (**B**) Annexin V-FITC/PI flow cytometry showing that NETs increased the apoptosis rate of HTR8/SVneo cells. (**C**) Western blot images showing NETs upregulated the protein expression of Bax/Bcl2 in HTR8/SVneo cells. * *p* < 0.05, ** *p* < 0.01.

To validate our bioinformatics findings, we collected placental tissues at delivery from women with GDM and healthy pregnant women. *MMP8* expression was elevated in GDM placentas, accompanied by increased mRNA levels of neutrophil markers (*CD11b* and *MPO*) (Figure 6A). In addition, immunohistochemical staining confirmed increased positivity for NET markers (citH3 and MPO) in GDM placentas (Figure 6B). HTR8/SVneo cells were used as an EVT model to evaluate the direct effects of NETs. Our results showed that both HG and NETs significantly inhibited proliferation (Figure 6C), migration (Figure 6D), and invasion (Figure 6E) of HTR8/SVneo cells. Moreover, NET treatment markedly increased intracellular ROS accumulation in HTR8/SVneo cells (Figure 7A). As illustrated in Figure 7B, the proportion of apoptotic cells was notably increased in the NET-exposed groups. Western blot analysis further revealed an increased Bax/Bcl-2 ratio (Figure 7C).

## 4. Discussion

**Figure 8:**
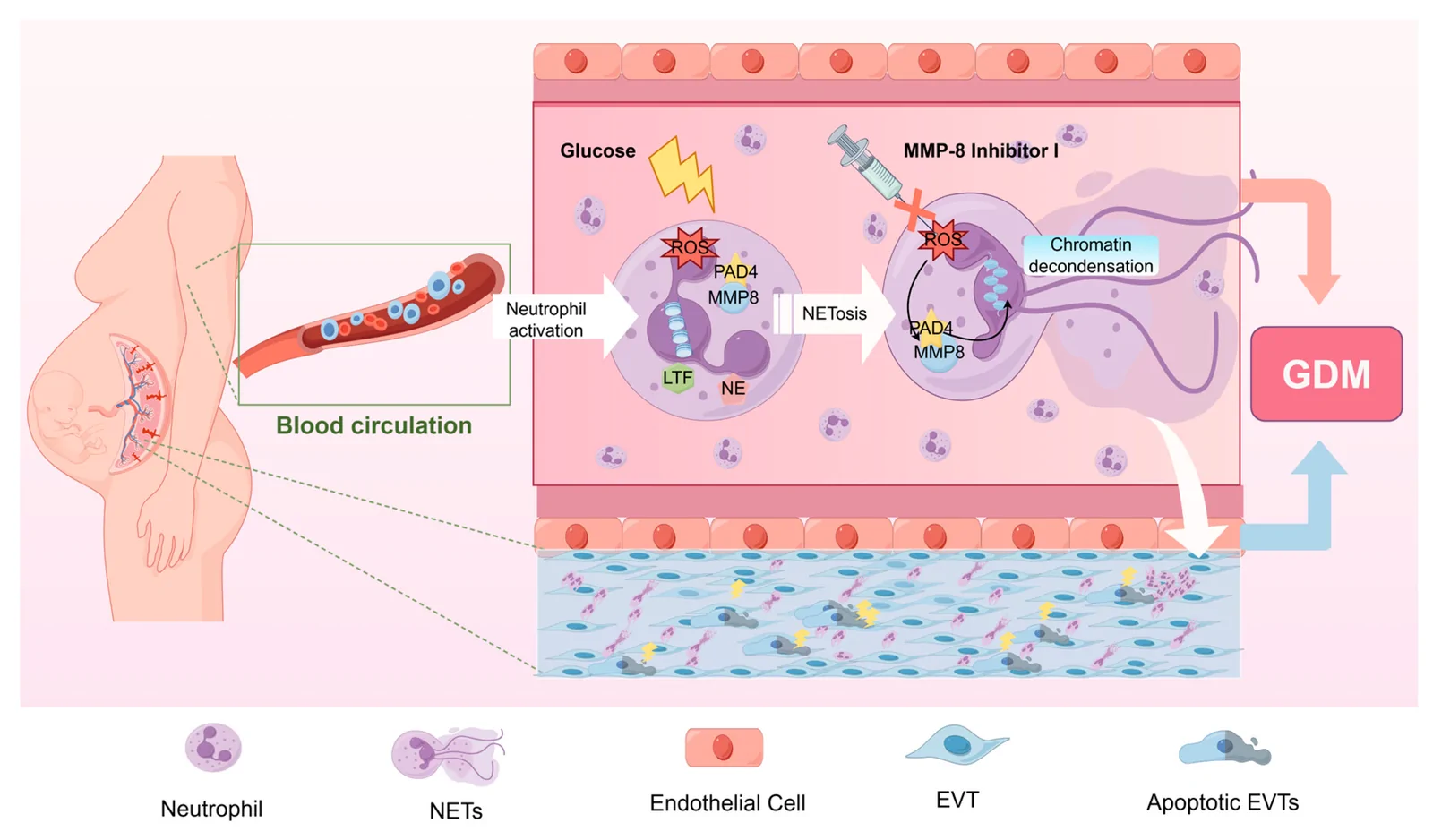
Schematic diagram of the potential molecular mechanism whereby MMP8-mediated NETosis contributes to placental injury in GDM. Hyperglycemia activates neutrophils, upregulating intracellular ROS, MMP8 and PADI4, which may subsequently promote chromatin decondensation and NETosis. NET accumulation in the placenta may impair EVT function and contribute to GDM-associated placental injury. Notably, MMP8 inhibitor 1 could reduce intracellular ROS production and attenuates NETosis. Abbreviations: NE, neutrophil elastase (ELANE-encoded protein); LTF, lactotransferrin; MMP8, matrix metalloproteinase 8; PAD4, peptidyl arginine deiminase 4 (PADI4-encoded protein); ROS, reactive oxygen species; EVT, extravillous trophoblast; NETs, neutrophil extracellular traps; GDM, gestational diabetes mellitus. This figure was generated by Figdraw (v 2.0).

Our study demonstrated that the inhibition of MMP8 could suppress intracellular ROS accumulation and attenuate NETosis in neutrophils. Furthermore, we identified a distinct placental neutrophil subpopulation with higher *PADI4* expression using scRNA-seq data from GDM women. Our findings suggest that MMP8, PADI4, and ROS, serving as the key intermediate mediators, are involved in GDM progression (Figure 8).

NETosis is tightly regulated by multiple pathways. Notably, ROS, acting as a vital inducer, initiates neutrophil PADI4-mediated histone citrullination and chromatin decondensation to promote NETosis [38,39]. NET accumulation has been reported in several metabolic disorders [14]. For instance, hyperglycemia and elevated homocysteine promote NETosis in type 2 diabetes mellitus (T2DM) by inducing neutrophil metabolic reprogramming and ROS production [40,41]. NETs contribute to diabetic complications by exacerbating macrophage inflammation and atherosclerotic lesions in diabetic mice [42]. Importantly, NETosis has also been implicated in pregnancy complications, particularly preeclampsia. Women with preeclampsia exhibited neutrophil hyperactivation and increased plasma DNA levels, accompanied by elevated NET-associated components (MPO and histones), indicating excessive NETosis [43]. GSK484, a PADI4 inhibitor, protects against preeclampsia in rats by suppressing PAD4-mediated placental NETosis, thereby alleviating maternal placental microvascular injury and improving pregnancy outcomes [44]. In the current study, we found that NETs were elevated in placental tissues from women with GDM, and plasma NETs were also increased following an oral glucose challenge. Our data were consistent with previous evidence that elevated glucose levels are associated with enhanced neutrophil activation and NETosis [45,46].

MMP8 is a highly active proteinase involved in the initiation of type I collagen degradation and extracellular matrix remodeling. *MMP8* expression was elevated in peripheral blood cfRNA from women with GDM [47]. Moreover, exosomal miR-26b-5p directly targets *MMP8* to mediate NETs, thereby promoting diabetic wound healing [48]. We observed increased expression of *MMP8*, and NETosis-related genes (*ELANE*, *PADI4*) in peripheral blood leukocytes from GDM. In addition, the expression of *MMP8* showed significant positive associations with *PADI4*. In primary human neutrophils, inhibition of MMP8 attenuated PMA-induced NETosis, accompanied by reduced expression of *PADI4* and *ELANE*. Co-IP assays further revealed a physical interaction between MMP8 and PADI4 proteins in neutrophils. Oxidative stress and inflammation are well-established core contributors to the initiation and progression of GDM [49,50]. In our study, inhibition of MMP8 attenuated PMA-induced intracellular ROS accumulation in neutrophils, while it upregulated the expression of antioxidant mediators, including *SOD2* and *GPX4.* These expression patterns suggest an association between *MMP8* expression and NETosis in GDM.

Neutrophils often exhibit a pro-inflammatory phenotype in chronic inflammatory diseases and further regulate inflammation and tissue remodeling through interactions with other cells [51]. Yang et al. conducted scRNA-seq profiling of placentas from patients with GDM and focused on the features of trophoblasts, NK cells and macrophages [52]. In our study, we specifically focused on the placental neutrophil population in GDM. The expression of *MMP8*, *LTF*, *ELANE*, and *PADI4* increased in placental neutrophils from GDM patients. Also, the expression of *MMP8* and neutrophil markers (*CD11b* and *MPO*) was elevated in our placental samples, indicating *MMP8* might be involved in neutrophil accumulation in GDM placentas. We further identified two distinct neutrophil subpopulations (Cluster 0 and Cluster 6) using scRNA-seq data. The DEGs of Cluster 6 were mainly enriched in inflammatory pathways and placental development, suggesting this subset might represent a potential effector population associated with placental dysfunction. Cell–cell communication analysis also revealed enhanced predicted interactions between Cluster 6 and EVTs in GDM placentas, mediated by ligand–receptor pairs such as NAMPT and FN1. Next, the HTR8/SVneo cell line was utilized to investigate the potential effects of NETs on placental function in vitro. NET exposure impaired the proliferation, migration and invasion of HTR8/SVneo cells, accompanied by increased apoptosis and ROS accumulation. These findings suggest that NET accumulation is associated with trophoblast dysfunction and may represent a potential mechanism linking neutrophil activation to placental injury in GDM.

Several limitations should be acknowledged. Although our findings suggest potential links among hyperglycemia, MMP8, NETosis, oxidative stress, and placental dysfunction in GDM, causal relationships remain to be established. Further validation through genetic manipulation approaches, in vivo animal models, and longitudinal clinical cohort studies is required to support future translational investigations. In addition, the limited cohort size may restrict the robustness of our clinical observations. The scRNA-seq and cell–cell communication analyses provide valuable insights into neutrophil heterogeneity and potential intercellular interactions; however, these findings remain observational and computationally inferred, requiring functional validation. Finally, although HTR8/SVneo cells provide a stable in vitro model, they may not fully recapitulate the characteristics of primary EVTs, and further studies using primary cells are warranted.

Our study collectively demonstrates that hyperglycemia is associated with increased *MMP8* expression and NETosis, while excessive NETosis is implicated in placental dysfunction in GDM. We identified MMP8 as a potential NETosis-associated molecule and a candidate target for future therapeutic exploration in GDM.

## Figures and Tables

**Table 1:** Baseline Characteristics of the Study Participants.

Characteristics	Control (*n* = 79)	GDM (*n* = 76)	Effect Size (95% CI)	*p* Value
Demographic characteristics				
Age (years)	31 (28, 34)	32 (29, 35)	1 (0 to 2)	0.063
Prepregnancy weight (kg)	58.00 (52.00, 64.00)	60.00 (54.25, 66.88)	3.00 (0.00 to 6.00)	0.018
Prepregnancy BMI (kg/m^2^)	22.19 ± 2.89	23.65 ± 3.87	1.46 (0.36 to 2.55)	0.010
Gravidity	1 (1, 2)	2 (1, 2)	0.00 (0.00 to 0.00)	0.060
Parity	0 (0, 0)	0 (0, 1)	0.00 (0.00 to 0.00)	0.341
Pregnancy outcomes				
Gestational weeks at delivery	39.00 (38.57, 39.46)	38.86 (38.14, 39.14)	−0.29 (−0.57 to 0.00)	0.039
Vaginal delivery (%)	24 (30.38)	20 (26.32)	−0.04 (−0.19 to 0.11)	0.575
Cesarean delivery (%)	55 (69.62)	56 (73.68)	0.04 (−0.11 to 0.19)	0.575
Weight-related parameters				
Weight at delivery (kg)	71.15 ± 8.62	72.95 ± 10.51	1.80 (−1.29 to 4.89)	0.251
BMI at delivery (kg/m^2^)	27.31 ± 3.23	27.87 ± 4.13	0.55 (−0.64 to 1.75)	0.362
Gestational weight gain (kg)	13.00 (10.00, 15.88)	10.00 (7.00, 14.00)	−3.00 (−4.00 to −1.00)	0.002
Glycemic parameters				
Fasting glucose (mmol/L)	4.55 (4.28, 4.81)	5.04 (4.75, 5.37)	0.51 (0.35 to 0.66)	<0.001
1 h OGTT glucose (mmol/L)	7.99 (6.82, 8.70)	10.20 (9.31, 10.97)	2.29 (1.75 to 2.87)	<0.001
2 h OGTT glucose (mmol/L)	7.00 (6.45, 7.98)	8.80 (7.94, 9.62)	1.75 (1.29 to 2.28)	<0.001
Peripartum Fasting glucose (mmol/L)	4.32 (3.87, 5.08)	4.52 (4.05, 5.54)	0.29 (−0.02 to 0.57)	0.061
Glycemic management strategies				
Diet and exercise only (%)	/	70 (92.11)	/	/
Metformin therapy (%)	/	1 (1.32)	/	/
Insulin therapy (%)	/	4 (5.26)	/	/
Metformin plus insulin therapy (%)	/	1(1.32)	/	/
Glycemic control status				
Poor glycemic control (%)	/	7 (9.21)	/	/
Pregnancy complications				
Gestational hypertension (%)	1 (1.27)	3 (3.95)	0.03 (−0.04 to 0.11)	0.361
Preeclampsia (%)	2 (2.53)	1 (1.32)	−0.01 (−0.08 to 0.06)	1.000
Pregnancy comorbidities				
Hypothyroidism (%)	7 (8.86)	3 (3.95)	−0.05 (−0.14 to 0.05)	0.328
Vaginal Group B Streptococcus colonization (%)	3 (3.80)	7 (9.21)	0.05 (−0.04 to 0.15)	0.204
Uterine fibroids (%)	8 (10.13)	9 (11.84)	0.02 (−0.09 to 0.13)	0.733
Antenatal medication exposure				
Antihypertensive medication (%)	2 (2.53)	3 (3.95)	0.01 (−0.06 to 0.10)	0.677
Levothyroxine (%)	5 (6.33)	3 (3.95)	−0.02 (−0.11 to 0.07)	0.720
Aspirin (%)	2 (2.53)	3 (3.95)	0.01 (−0.06 to 0.10)	0.677
Neonatal characteristics				
Male sex (%)	36 (45.57)	41 (53.95)	0.08 (−0.08 to 0.24)	0.297
Birth weight (g)	3240 (2990, 3520)	3230 (3010, 3560)	15 (−110 to 150)	0.828
Birth length (cm)	50 (50, 51)	50 (50, 51)	0 (0 to 1)	0.185
Birth head circumference (cm)	34 (33, 34)	34 (33, 34)	0 (0 to 0)	0.658
Blood glucose (mmol/L)	3.70 (3.20, 4.53)	3.55 (3.13, 4.20)	−0.30 (−0.50 to 0.00)	0.048
Transcutaneous bilirubin, forehead (µmol/L)	97.73 ± 19.14	103.20 ± 19.72	5.43 (−0.76 to 11.61)	0.085
Transcutaneous bilirubin, chest (µmol/L)	92.04 ± 23.32	98.13 ± 20.20	6.09 (−0.86 to 13.05)	0.085
Neonatal outcomes				
Macrosomia (%)	1 (1.27)	3 (3.95)	0.03 (−0.04 to 0.11)	0.361
Fetal distress (%)	3 (3.80)	1 (1.32)	−0.02 (−0.10 to 0.05)	0.620

Data are presented as mean ± standard deviation (SD) for normally distributed continuous variables, and median (interquartile range, IQR) for non-normally distributed continuous variables. Normally distributed variables were compared using independent samples *t*-test, and non-normally distributed variables were compared using Mann–Whitney U test. Chi-square test was used for categorical variables with adequate cell counts, while Fisher’s exact test was applied for indicators with small sample sizes in subgroups. Effect size with 95% confidence interval (95% CI) and two-sided *p* values were calculated for all between-group comparisons. *p* < 0.05 was considered statistically significant. The symbol “/” indicates indicators not applicable to the control group. OGTT, oral glucose tolerance test.

## Data Availability

Bulk RNA-Seq data used in this study were obtained from the Gene Expression Omnibus (GEO) database with accession number GSE92772 (https://identifiers.org/geo:GSE92772, accessed on 30 December 2024). scRNA-Seq data were retrieved from GEO under accession number GSE267340 (https://identifiers.org/geo:GSE267340, accessed on 11 October 2025).

## Supplementary Materials

The following supporting information can be downloaded at: https://www.mdpi.com/article/10.3390/antiox15080955/s1, Figure S1: Clustering tree across different resolutions, Figure S2: Dot plot of marker genes for cell type identification, Figure S3: Schematic diagram depicting the procedure of sample collection, Figure S4: Schematic diagram illustrating the treatment protocol for primary neutrophils, Figure S5: Identification of candidate hub genes in GDM peripheral blood, Figure S6: The morphology of primary neutrophil in response to different time and concentrations of exogenous NETs exposure, Figure S7: Relative mRNA expression levels of MMP1 and MMP9 in PMA-stimulated neutrophils with or without MMP8 inhibitor (M8I) treatment, Figure S8: Enrichment plots of downregulated DEGs in GDM placental neutrophils, Figure S9: Enrichment plots of downregulated DEGs in Cluster 6, Figure S10: Cell-cell communication network and EVT-neutrophil ligand-receptor crosstalk in control and GDM placentas; Table S1: Primers used for qPCR, Table S2: Comparison of laboratory parameters between healthy pregnant controls and women with GDM.

## Abbreviations

NETs	Neutrophil extracellular traps
GDM	Gestational diabetes mellitus
scRNA-seq	Single-cell transcriptomic sequencing
NETosis	NETs formation
OGTT	Oral glucose tolerance test
ROS	Reactive oxygen species
EVTs	Extravillous trophoblasts
DEGs	Differential expressed genes
PPI	Protein–protein interaction
GSEA	Gene set enrichment analysis
IADPSG	International Association of Diabetes and Pregnancy Study Groups
PMA	Phorbol 12-myristate 13-acetate
ELISA	Enzyme-linked immunosorbent assay
qPCR	Quantitative real-time PCR
Co-IP	Co-immunoprecipitation
IF	Immunofluorescence
IHC	Immunohistochemistry
HG	High glucose
LG	Low glucose
M8I	MMP8 inhibitor 1
CCK-8	Cell Counting Kit-8
HGB	Hemoglobin
MCH	Mean corpuscular hemoglobin
FIB	Fibrinogen
APTT	Activated partial thromboplastin time
UA	Uric acid
BUN	Blood urea nitrogen
OGTT 2 h glucose levels	2 h plasma glucose levels based on an OGTT
citH3	Citrullinated histone H3
UMAP	Uniform Manifold Approximation and Projection
VCTs	Villous cytotrophoblasts
SCTs	Syncytiotrophoblasts

## References

[1] Lam E.L., Walker J.M., Wang M.C., Venkatesh K.K., Khan S.S., Shah N.S. (2025). Gestational Diabetes in the US From 2016 to 2024. JAMA Intern. Med..

[2] Mercado-Evans V., Mejia M.E., Zulk J.J., Ottinger S., Hameed Z.A., Serchejian C., Marunde M.G., Robertson C.M., Ballard M.B., Ruano S.H. (2024). Gestational diabetes augments group B Streptococcus infection by disrupting maternal immunity and the vaginal microbiota. Nat. Commun..

[3] Tanaka T., Wada T., Uno K., Ogihara S., Ie H., Okekawa A., Ishikawa A., Ito T., Miyazawa Y., Sameshima A. (2021). Oestrogen receptor α in T cells controls the T cell immune profile and glucose metabolism in mouse models of gestational diabetes mellitus. Diabetologia.

[4] Cai M., Jiang X., Xu X., Zhao S., Sun Y., Yang Y., Yang P., Fang C., Huang Y. (2025). Integrating adipsin with novel cardiometabolic and inflammatory indices for enhanced early prediction of gestational diabetes mellitus: A prospective cohort study. Cardiovasc. Diabetol..

[5] Pinto Y., Frishman S., Turjeman S., Eshel A., Nuriel-Ohayon M., Shrossel O., Ziv O., Walters W., Parsonnet J., Ley C. (2023). Gestational diabetes is driven by microbiota-induced inflammation months before diagnosis. Gut.

[6] Huang S., Chen J., Cui Z., Ma K., Wu D., Luo J., Li F., Xiong W., Rao S., Xiang Q. (2023). Lachnospiraceae-derived butyrate mediates protection of high fermentable fiber against placental inflammation in gestational diabetes mellitus. Sci. Adv..

[7] Fang J., Wu X., He J., Zhang H., Chen X., Zhang H., Novakovic B., Qi H., Yu X. (2023). RBM15 suppresses hepatic insulin sensitivity of offspring of gestational diabetes mellitus mice via m6A-mediated regulation of CLDN4. Mol. Med..

[8] Yao T., Xiong Y., Hu Q., Chen Y., Li D., Yan J., Yang J., Wang Y., Cao H., Zhang F. (2026). Maternal gestational diabetes mellitus leads to adverse growth patterns and disease risk in offspring in vivo: Evidence from cross-generational effects on gut microbiota. BMC Microbiol..

[9] Yu W., Li Y., Jiang S., Tian J., Sun S.W., Zhang L., Xiao D. (2026). FTO-dependent m6A methylation mediates gestational diabetes mellitus-induced offspring cardiac senescent hypertrophy and dysfunction. iScience.

[10] Mutua V., Gershwin L.J. (2021). A Review of Neutrophil Extracellular Traps (NETs) in Disease: Potential Anti-NETs Therapeutics. Clin. Rev. Allergy Immunol..

[11] Shahzad A., Ni Y., Yang Y., Liu W., Teng Z., Bai H., Liu X., Sun Y., Xia J., Cui K. (2025). Neutrophil Extracellular Traps (NETs) in health and disease. Mol. Biomed..

[12] Schommer N., Gendron N., Krauel K., Van Bruggen S., Jarrot P.A., Maier A., Chan W., Langer H.F., Duerschmied D., Westermann D. (2025). Neutrophil extracellular traps and peptidylarginine deiminase 4-mediated inflammasome activation link diabetes to cardiorenal injury and heart failure. Eur. Heart J..

[13] Xia Y., Lan J., Yang J., Yuan S., Xie X., Du Q., Du H., Nie W., Jiang B., Zhao L. (2025). Saturated fatty acid-induced neutrophil extracellular traps contribute to exacerbation and biologic therapy resistance in obesity-related psoriasis. Cell Mol. Immunol..

[14] Wang H., Kim S.J., Lei Y., Wang S., Wang H., Huang H., Zhang H., Tsung A. (2024). Neutrophil extracellular traps in homeostasis and disease. Signal Transduct. Target. Ther..

[15] Fei Y., Huang X., Ning F., Qian T., Cui J., Wang X., Huang X. (2024). NETs induce ferroptosis of endothelial cells in LPS-ALI through SDC-1/HS and downstream pathways. Biomed. Pharmacother..

[16] Qi Y., Chen L., Ding S., Shen X., Wang Z., Qi H., Yang S. (2024). Neutrophil extracellular trap-induced ferroptosis promotes abdominal aortic aneurysm formation via SLC25A11-mediated depletion of mitochondrial glutathione. Free Radic. Biol. Med..

[17] Jia H., Yue G., Li P., Peng R., Jin R., Chen Y., Cao H., Yang K., Zhang X., Yi X. (2025). Neutrophil extracellular traps license macrophage production of chemokines to facilitate CD8+ T cell infiltration in obstruction-induced renal fibrosis. Protein Cell.

[18] Lin X., Zhang Y., He X., Chen Y., Chen N., Liu J., Wang M., Li Y., Yang H., Fan L. (2021). The Choline Metabolite TMAO Inhibits NETosis and Promotes Placental Development in GDM of Humans and Mice. Diabetes.

[19] Shen D., Lu Y., Li G., Hu M., Li S., Ju H., Zhang M., Wang X. (2021). Mechanism of neutrophil extracellular traps generation and their role in trophoblasts apoptosis in gestational diabetes mellitus. Cell Signal.

[20] Stoikou M., Grimolizzi F., Giaglis S., Schäfer G., van Breda S.V., Hoesli I.M., Lapaire O., Huhn E.A., Hasler P., Rossi S.W. (2017). Gestational Diabetes Mellitus Is Associated with Altered Neutrophil Activity. Front. Immunol..

[21] Ounadjela J.R., Zhang K., Kobayashi-Kirschvink K.J., Jin K., Russell A.J.C., Lackner A.I., Callahan C., Viggiani F., Dey K.K., Jagadeesh K. (2024). Spatial multiomic landscape of the human placenta at molecular resolution. Nat. Med..

[22] Luo F., Liu F., Guo Y., Xu W., Li Y., Yi J., Fournier T., Degrelle S., Zitouni H., Hernandez I. (2023). Single-cell profiling reveals immune disturbances landscape and HLA-F-mediated immune tolerance at the maternal-fetal interface in preeclampsia. Front. Immunol..

[23] Bert S., Ward E.J., Nadkarni S. (2021). Neutrophils in pregnancy: New insights into innate and adaptive immune regulation. Immunology.

[24] Derisoud E., Jiang H., Zhao A., Chavatte-Palmer P., Deng Q. (2024). Revealing the molecular landscape of human placenta: A systematic review and meta-analysis of single-cell RNA sequencing studies. Hum. Reprod. Update.

[25] Levenson D., Romero R., Miller D., Galaz J., Garcia-Flores V., Neshek B., Pique-Regi R., Gomez-Lopez N. (2025). The maternal-fetal interface at single-cell resolution: Uncovering the cellular anatomy of the placenta and decidua. Am. J. Obstet. Gynecol..

[26] Zhou Z., Yang X. (2024). An update review of the application of single-cell RNA sequencing in pregnancy-related diseases. Front. Endocrinol..

[27] Hao Y., Stuart T., Kowalski M.H., Choudhary S., Hoffman P., Hartman A., Srivastava A., Molla G., Madad S., Fernandez-Granda C. (2024). Dictionary learning for integrative, multimodal and scalable single-cell analysis. Nat. Biotechnol..

[28] Germain P.L., Lun A., Garcia Meixide C., Macnair W., Robinson M.D. (2021). Doublet identification in single-cell sequencing data using scDblFinder. F1000Res.

[29] Wang Z., Yang S., Koga Y., Corbett S.E., Shea C.V., Johnson W.E., Yajima M., Campbell J.D. (2022). Celda: A Bayesian model to perform co-clustering of genes into modules and cells into subpopulations using single-cell RNA-seq data. NAR. Genom. Bioinform..

[30] Street K., Risso D., Fletcher R.B., Das D., Ngai J., Yosef N., Purdom E., Dudoit S. (2018). Slingshot: Cell lineage and pseudotime inference for single-cell transcriptomics. BMC Genom..

[31] Jin S., Guerrero-Juarez C.F., Zhang L., Chang I., Ramos R., Kuan C.H., Myung P., Plikus M.V., Nie Q. (2021). Inference and analysis of cell-cell communication using CellChat. Nat. Commun..

[32] Metzger B.E., Gabbe S.G., Persson B., Buchanan T.A., Catalano P.A., Damm P., Dyer A.R., Leiva A., Hod M., Kitzmiler J.L. (2010). International association of diabetes and pregnancy study groups recommendations on the diagnosis and classification of hyperglycemia in pregnancy. Diabetes Care.

[33] Reis L.R., Nascimento R.O., Massafera M.P., Di Mascio P., Ronsein G.E. (2025). Investigating neutrophil responses to stimuli: Comparative analysis of reactive species-dependent and independent mechanisms. Redox Biol..

[34] Wang S., Song Y., Wang Z., Chang X., Wu H., Yan Z., Wu J., He Z., Kang L., Hu W. (2024). Neutrophil-derived PAD4 induces citrullination of CKMT1 exacerbates mucosal inflammation in inflammatory bowel disease. Cell Mol. Immunol..

[35] Mortaz E., Adcock I.M., Ito K., Kraneveld A.D., Nijkamp F.P., Folkerts G. (2010). Cigarette smoke induces CXCL8 production by human neutrophils via activation of TLR9 receptor. Eur. Respir. J..

[36] Martyn K.D., Kim M.J., Quinn M.T., Dinauer M.C., Knaus U.G. (2005). p21-activated kinase (Pak) regulates NADPH oxidase activation in human neutrophils. Blood.

[37] Belliveau N.M., Footer M.J., Akdoǧan E., van Loon A.P., Collins S.R., Theriot J.A. (2023). Whole-genome screens reveal regulators of differentiation state and context-dependent migration in human neutrophils. Nat. Commun..

[38] Azzouz D., Palaniyar N. (2024). How Do ROS Induce NETosis? Oxidative DNA Damage, DNA Repair, and Chromatin Decondensation. Biomolecules.

[39] Liu C., Chen Q., Chen W., Han X., Qiao X., Guo J., Su W., Dai Q. (2026). IL-17A Promotes NETs Formation via the PKCζ-ERK-ROS-PAD4 Pathway in a Mouse Model of Ischemic Stroke. CNS Neurosci. Ther..

[40] Joshi M.B., Ahamed R., Hegde M., Nair A.S., Ramachandra L., Satyamoorthy K. (2020). Glucose induces metabolic reprogramming in neutrophils during type 2 diabetes to form constitutive extracellular traps and decreased responsiveness to lipopolysaccharides. Biochim. Biophys. Acta Mol. Basis Dis..

[41] Joshi M.B., Baipadithaya G., Balakrishnan A., Hegde M., Vohra M., Ahamed R., Nagri S.K., Ramachandra L., Satyamoorthy K. (2016). Elevated homocysteine levels in type 2 diabetes induce constitutive neutrophil extracellular traps. Sci. Rep..

[42] Josefs T., Barrett T.J., Brown E.J., Quezada A., Wu X., Voisin M., Amengual J., Fisher E.A. (2020). Neutrophil extracellular traps promote macrophage inflammation and impair atherosclerosis resolution in diabetic mice. JCI Insight.

[43] Hu Y., Li H., Yan R., Wang C., Wang Y., Zhang C., Liu M., Zhou T., Zhu W., Zhang H. (2018). Increased Neutrophil Activation and Plasma DNA Levels in Patients with Pre-Eclampsia. Thromb. Haemost..

[44] Li Y.Y., Zhang Z.Y., Wang J., Wang D.P., Zhao W.F., Ma H.J., Lin M.M., Cao J.M., Han J.B. (2026). GSK484 attenuates placental endothelial injury and improves gestation outcome in rats with preeclampsia by inhibiting PAD4-mediated neutrophil extracellular traps formation. Eur. J. Pharmacol..

[45] Wang Q., Lin W., Lei K., Wang H., Zhang X., Jiang S., Zhang D., Wang W., Cao S., Li Y. (2024). Hyperglycemia-Enhanced Neutrophil Extracellular Traps Drive Mucosal Immunopathology at the Oral Barrier. Adv. Sci..

[46] Chu Z., Huang Q., Ma K., Liu X., Zhang W., Cui S., Wei Q., Gao H., Hu W., Wang Z. (2023). Novel neutrophil extracellular trap-related mechanisms in diabetic wounds inspire a promising treatment strategy with hypoxia-challenged small extracellular vesicles. Bioact. Mater..

[47] Del Vecchio G., Li Q., Li W., Thamotharan S., Tosevska A., Morselli M., Sung K., Janzen C., Zhou X., Pellegrini M. (2021). Cell-free DNA Methylation and Transcriptomic Signature Prediction of Pregnancies with Adverse Outcomes. Epigenetics.

[48] Rui S., Dai L., Zhang X., He M., Xu F., Wu W., Armstrong D.G., You Y., Xiao X., Ma Y. (2024). Exosomal miRNA-26b-5p from PRP suppresses NETs by targeting MMP-8 to promote diabetic wound healing. J. Control Release.

[49] Saucedo R., Ortega-Camarillo C., Ferreira-Hermosillo A., Díaz-Velázquez M.F., Meixueiro-Calderón C., Valencia-Ortega J. (2023). Role of Oxidative Stress and Inflammation in Gestational Diabetes Mellitus. Antioxidants.

[50] Buczyńska-Backiel A., Kościuszko M., Krętowski A.J., Szelachowska M. (2026). Assessment of the impact of oxidative stress and inflammation on placental angiogenesis in women with gestational diabetes mellitus (GDM). Rev. Endocr. Metab. Disord..

[51] Torfs K., Vermeersch G., Gouwy M., Devos T., Proost P., Struyf S. (2026). Neutrophils as critical orchestrators of chronic inflammation. Cell Mol. Immunol..

[52] Yang Y., Guo F., Peng Y., Chen R., Zhou W., Wang H., OuYang J., Yu B., Xu Z. (2021). Transcriptomic Profiling of Human Placenta in Gestational Diabetes Mellitus at the Single-Cell Level. Front. Endocrinol..

