# Supplementary material for: MMP8 Promotes NETosis in Gestational Diabetes Mellitus
Source: Antioxidants (Basel). 2026 Jul 30;15(8):955. doi: 10.3390/antiox15080955 (PMC13509984; doi:10.3390/antiox15080955)
Supplement: Supplementary file 1 [file antioxidants-15-00955-s001.zip › antioxidants-4386878-Supplementary Materials.pdf]

Supplementary Information

MMP8 Promotes NETosis in Gestational Diabetes Mellitus

The supplementary file includes 2 Tables and 10 Figures.

Supplementary Table S1. Primers used for qPCR.

Gene symbols	Gene accession numbers	Primers (5'-3')
ACTB	NM_001101.5	F: CACCATTGGCAATGAGCGGTTC
		R: AGGTCTTTGCGGATGTCCACGT
MMP8	NM_002424.3	F: CAACCTACTGGACCAAGCACAC
		R: TGTAGCTGAGGATGCCTTCTCC
LTF	NM_002343.6	F: GGCTACTTCACTGCCATCCAGA
		R: ACTCCACTGGTTACACTTGCGC
ABCA13	NM_152701.5	F: GTACGTGTGCTCACCATCGTTG
		R: AAGTGGTCCTGCGCCAAAGTGA
ELANE	NM_001972.4	F: TGCGCCCAACTTCGTCATGTCG
		R: CGTAGCCGTTTTCGAAGATGCG
PADI4	NM_012387.3	F: GCACAACATGGACTTCTACGTGG
		R: CACGCTGTCTTGGAACACCACACA
SOD1	NM_000454.5	F: CTCACTCTCAGGAGACCATTGC
		R: CCACAAGCCAAACGACTTCCAG
SOD2	NM_000636.4	F: CTGGACAAACCTCAGCCCTAAC
		R: AACCTGAGCCTTGGACACCAAC
GPX1	NM_000581.4	F: GTGCTCGGCTTCCCGTGCAAC
		R: CTCGAAGAGCATGAAGTTGGGC
GPX4	NM_002085.5	F: ACAAGAACGGCTGCGTGGTGAA
		R: GCCACACACTTGTGGAGCTAGA
CD11b	NM_000632.4	F: GGAACGCCATTGTCTGCTTTCG
		R: ATGCTGAGGTCATCCTGGCAGA
MPO	NM_000250.2	F: GAGCAGGACAAATACCGCACCA
		R: AGAGAAGCCGTCCTCATACTCC

Supplementary Table S2. Comparison of laboratory parameters between healthy pregnant controls and women with GDM

**Characteristics**	**Control (*n*=79)**	**GDM (*n*=76)**	**Effect size (95%CI)**	***p* value**
HGB	121.70±11.24	125.40±10.87	3.65 (0.13 to 7.17)	0.042
MCH	30.87±2.14	31.55±1.86	0.68 (0.04 to 1.32)	0.037
MCHC	336.2±6.64	338.4±6.89	2.21 (0.05 to 4.36)	0.045
D-dimer	1482 (1150, 2102)	1870 (1403, 2415)	275 (46 to 506)	0.019
PTA	110 (103, 116)	113 (107, 118)	3 (0 to 6)	0.016
PT	10.30 (10.00, 10.70)	10.10 (9.90, 10.40)	-0.20 (-0.30 to 0.00)	0.014
INR	0.94 (0.91, 0.98)	0.93 (0.90, 0.95)	-0.02 (-0.03 to 0.00)	0.010
ALT	9 (8, 12)	10 (9, 14)	1 (0 to 2)	0.033
GGT	8.00 (5.00, 11.00)	9.00 (6.00, 13.75)	1.00 (0.00 to 3.00)	0.048

Data are presented as mean ± standard deviation (SD) for normally distributed continuous variables, and median (interquartile range, IQR) for non-normally distributed continuous variables. Normally distributed variables were compared using the independent samples t-test, and non-normally distributed variables were compared using the Mann-Whitney U test. Effect sizes with 95% confidence interval (95% CI) and two-sided *P* values were calculated for all between-group comparisons. $P < 0.05$ was considered statistically significant. HGB, hemoglobin; MCH, mean corpuscular hemoglobin; MCHC, mean corpuscular hemoglobin concentration; PT, prothrombin time; PTA, prothrombin time activity; INR, international normalized ratio; ALT, alanine aminotransferase; GGT, gamma-glutamyl transferase

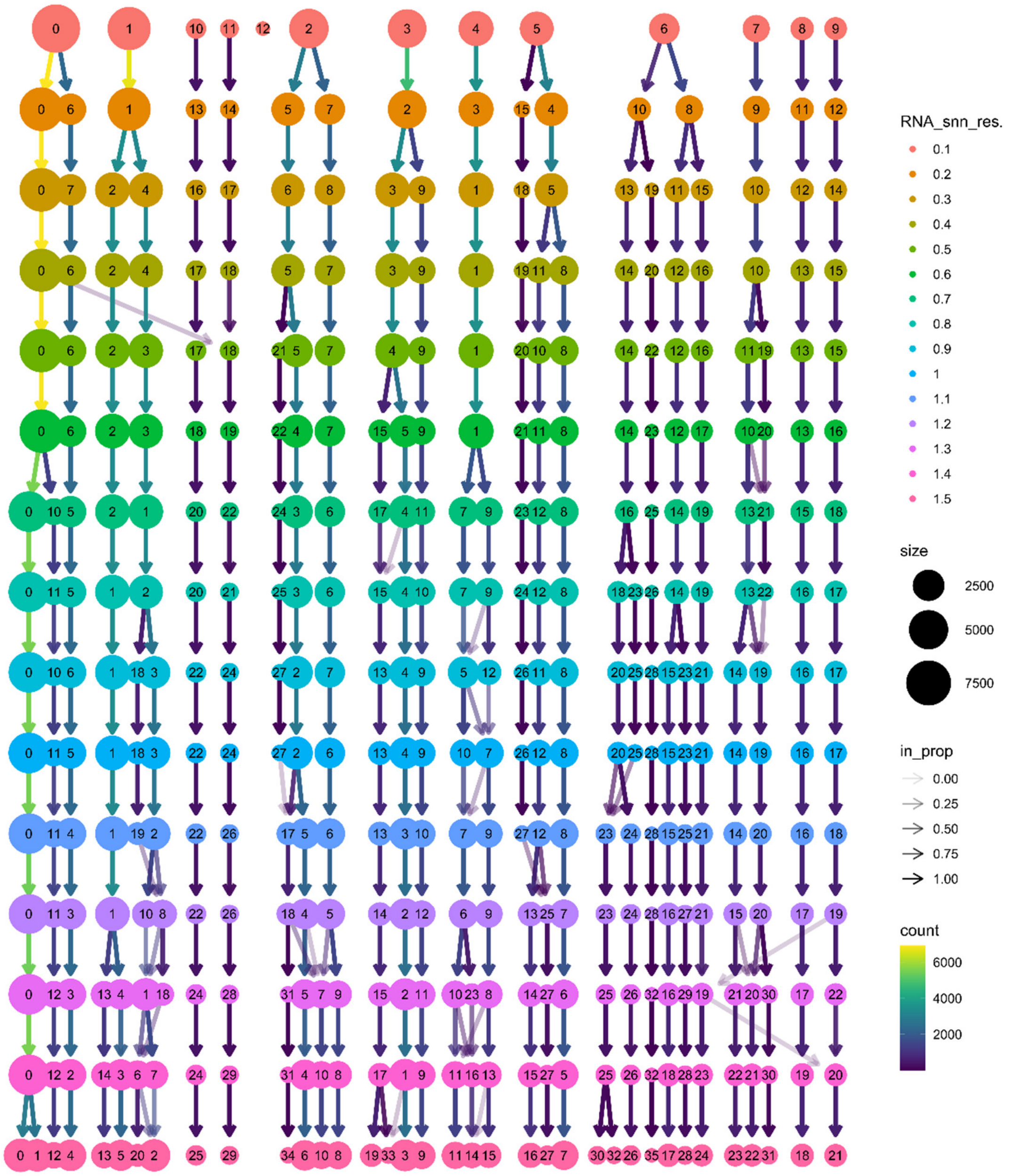

Supplementary Figure S1. Clustering tree across different resolutions. The clustering tree visualizes the relationships between cell clusters as the resolution parameter increases from 0.1 to 1.5. Each node (circle) represents a cluster at a specific resolution, with the size of the node proportional to the number of cells within that cluster. The edges (arrows) between nodes indicate cells moving between clusters as the resolution increases. The color and transparency of the edges represent the proportion of cells coming from the parent cluster, where darker and thicker arrows indicate higher stability.

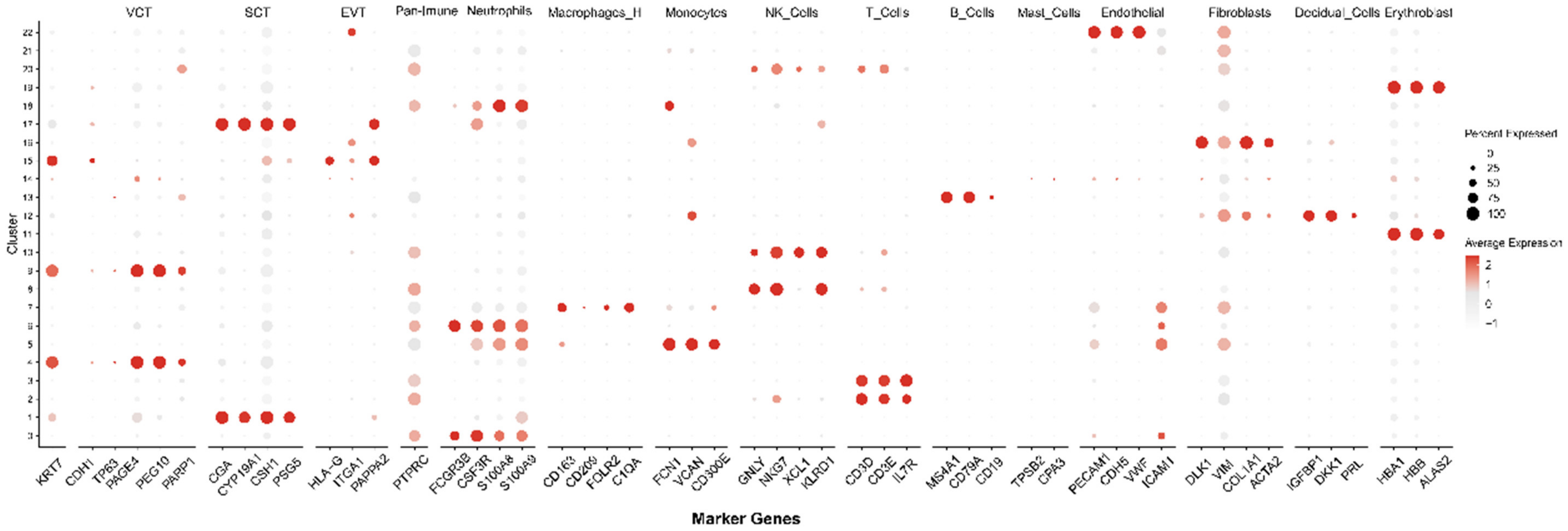

Supplementary Figure S2. Dot plot of marker genes for cell type identification. The dot plot displays the expression patterns of selected marker genes across clusters. The size of each dot represents the percentage of cells within a cluster expressing the gene (Percent Expressed), and the color intensity reflects the scaled average expression level (Average Expression), ranging from low (white) to high (red).

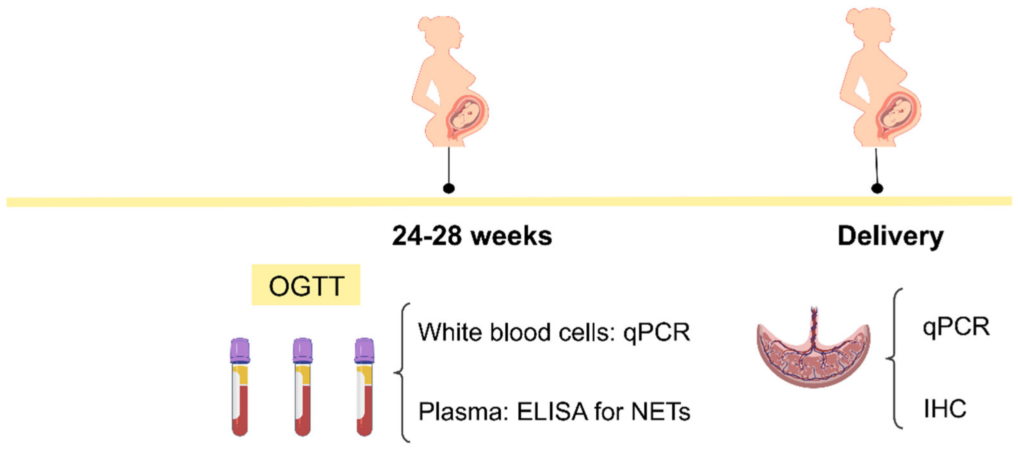

Supplementary Figure S3. Schematic diagram depicting the procedure of sample collection. OGTT, Oral glucose tolerance test. This figure was generated by Medpeer.

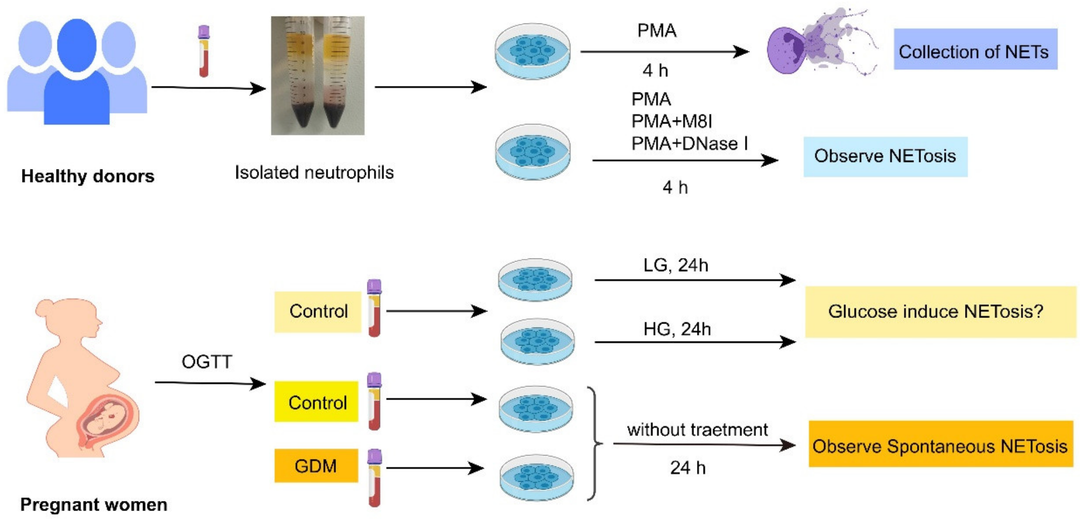

Supplementary Figure S4. Schematic diagram illustrating the treatment protocol for primary neutrophils. OGTT, oral glucose tolerance test; PMA, phorbol 12-myristate 13-acetate; M8I, MMP8 inhibitor-1; DNase I, Deoxyribonuclease I; LG, low glucose (5.5 mmol/L); HG, high glucose (33.3mmol/L). This figure was generated by Medpeer.

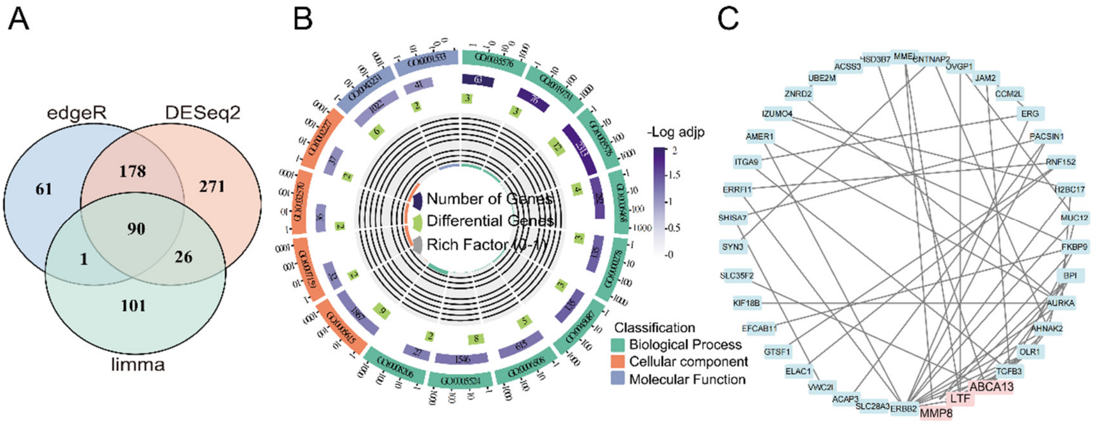

Supplementary Figure S5. Identification of candidate hub genes in GDM peripheral blood. (A) Venn diagram displaying shared DEGs identified by edgeR, DESeq2, and limma. (B) GO enrichment analysis of shared DEGs. (C) protein–protein interaction (PPI) network of shared DEGs.

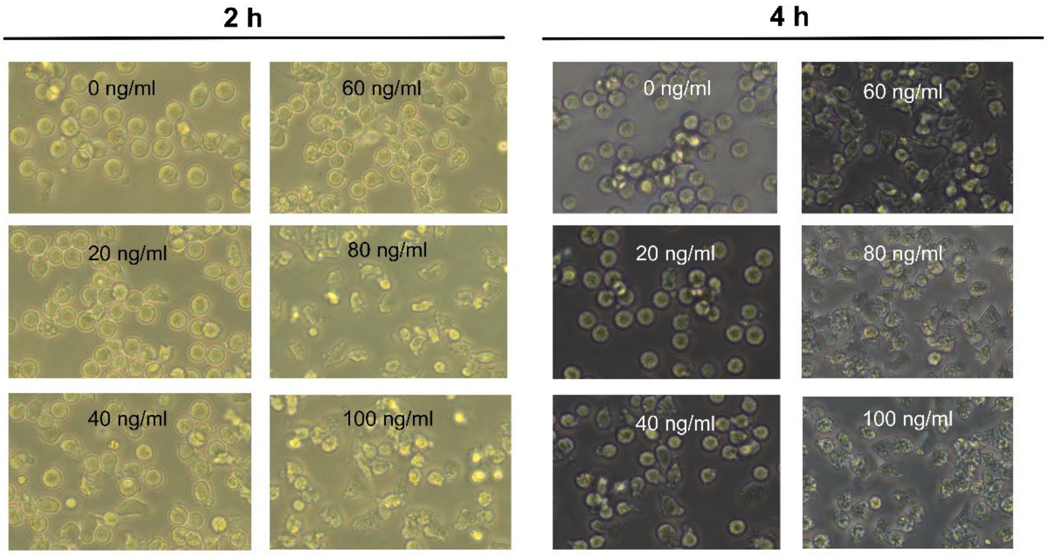

Supplementary Figure S6. The morphology of primary neutrophil in response to different time and concentrations of exogenous NETs exposure.

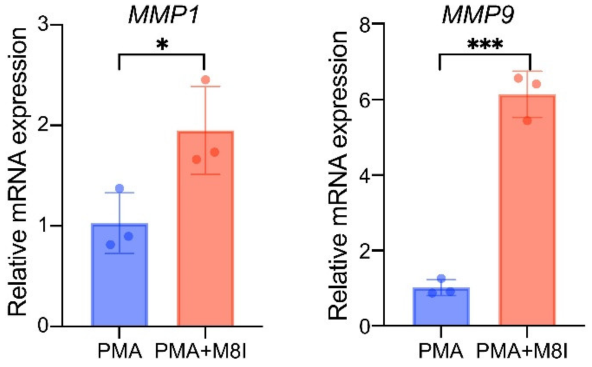

Supplementary Figure S7. Relative mRNA expression levels of *MMP1* and *MMP9* in PMA-stimulated neutrophils with or without MMP8 inhibitor (M8I) treatment.

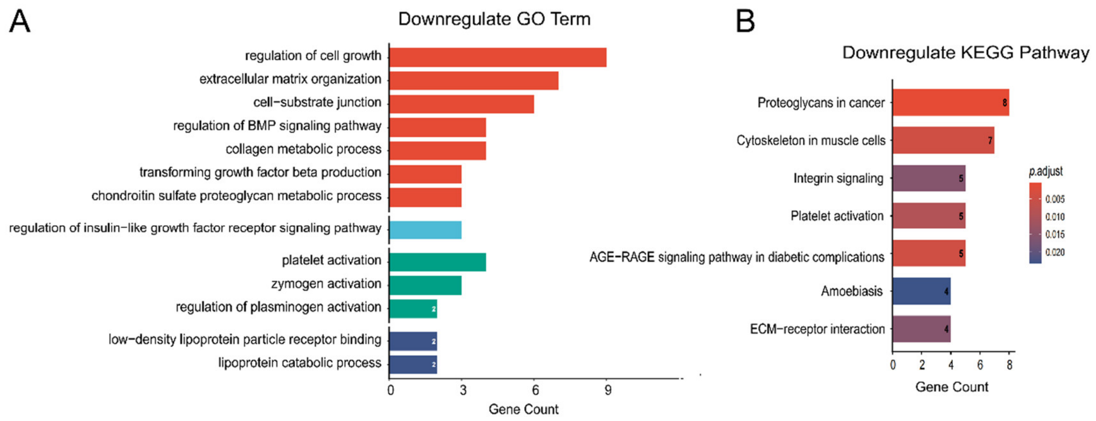

Supplementary Figure S8. Enrichment plots of downregulated DEGs in GDM placental neutrophils.

(A) GO terms of downregulated differentially expressed gene (DEGs) in GDM placental neutrophils.

(B) KEGG pathways of downregulated DEGs in GDM placental neutrophils.

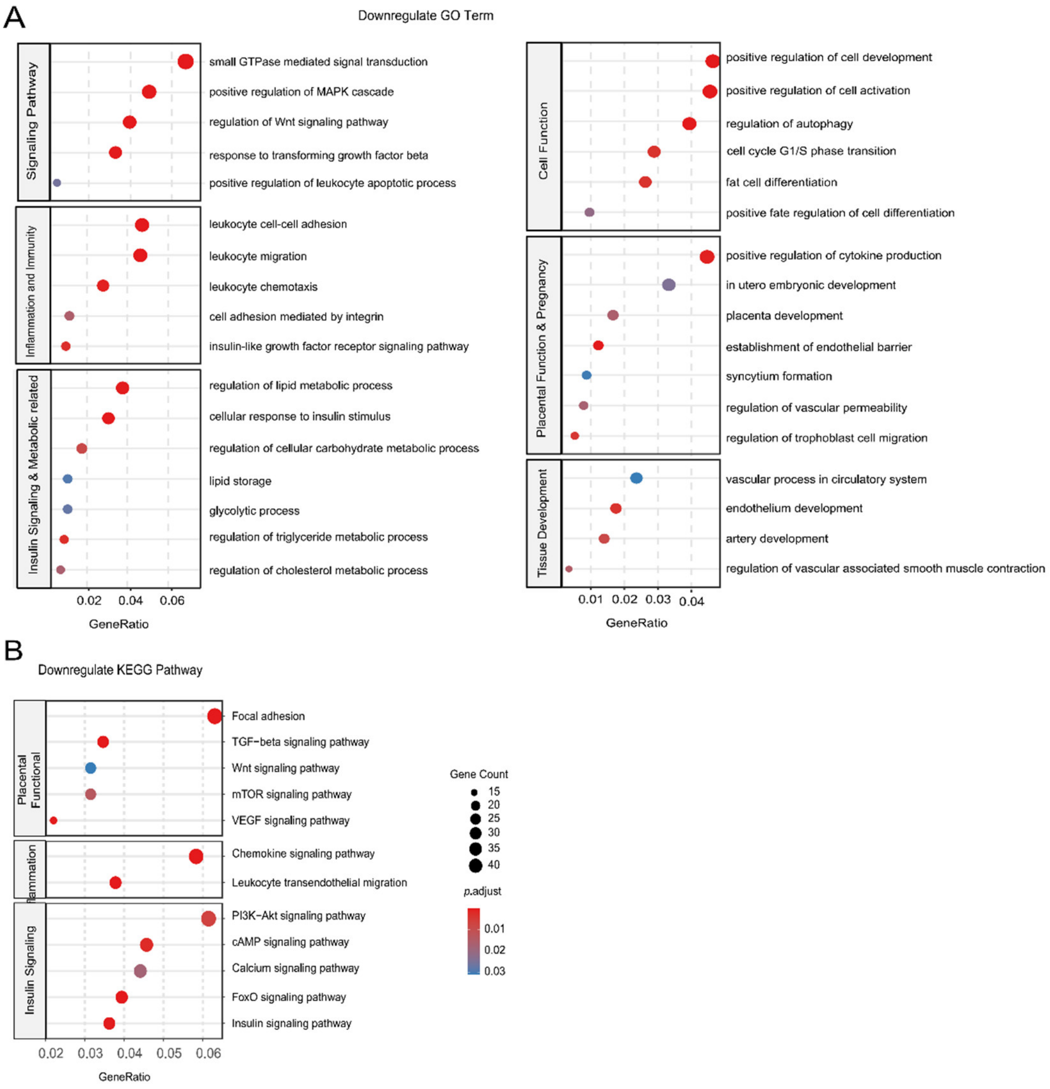

Supplementary Figure S9. Enrichment plots of downregulated DEGs in Cluster 6.

(A) GO terms of downregulated differentially expressed gene (DEGs) in Cluster 6. (B) KEGG pathways of downregulated DEGs in Cluster 6.

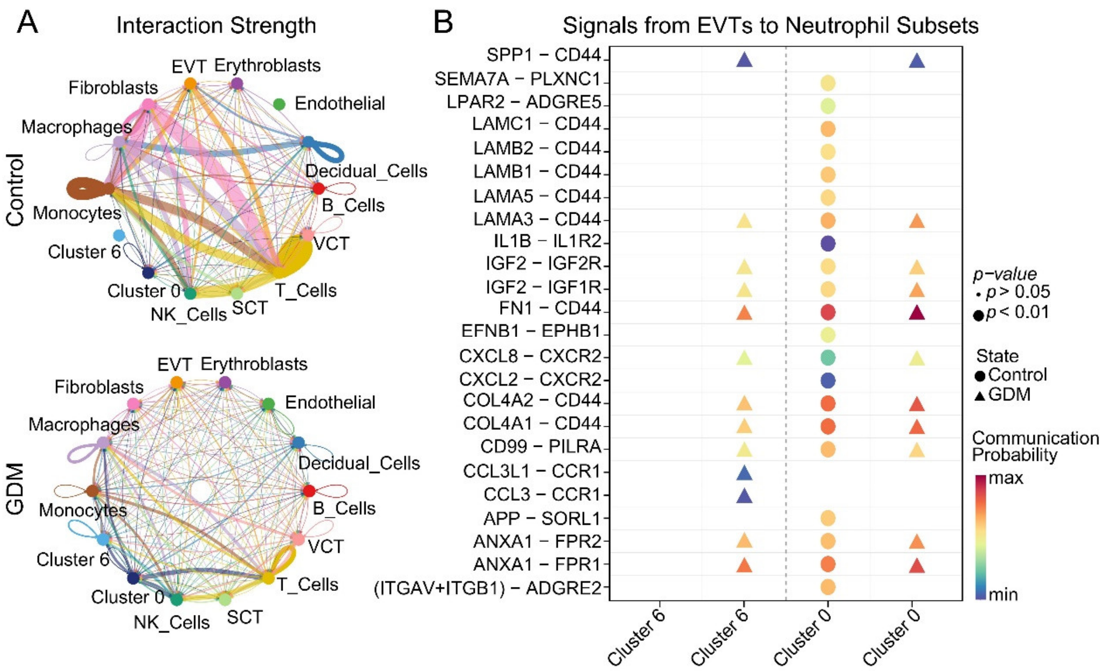

Supplementary Figure S10. Cell-cell communication network and EVT-neutrophil ligand-receptor crosstalk in control and GDM placentas. (A) cell-cell interaction strength network of placental cells in control and GDM groups. (B) Ligand-receptor communication probability of signals secreted by EVTs to two neutrophil subclusters (Cluster 0, Cluster 6).
